# Phenotypic Response Surfaces–Guided Optimization (PRS-OPT) of Propolis-Metformin-Regorafenib Combination Therapy for MASLD-Associated Hepatocellular Carcinoma

**DOI:** 10.32604/or.2026.074145

**Published:** 2026-05-21

**Authors:** Yi-Sian Huang, Chung-Yung Ma, Hsiao-Yuh Roan, Cheng-Hsiung Chiang, Hsiao-Hui Tsou, Chen-Hui Chen, Yi-Fan Lin, Horng-Dar Wang, Chiou-Hwa Yuh

**Affiliations:** 1 Institute of Molecular and Genomic Medicine, National Health Research Institutes, Zhunan, Miaoli, Taiwan; 2 Institute of Biotechnology, National Tsing Hua University, Hsinchu, Taiwan; 3 Institute of Cellular and Organismic Biology, Academia Sinica, Nankang, Taipei, Taiwan; 4 Institute of Population Health Sciences, National Health Research Institutes, Zhunan, Miaoli, Taiwan; 5 Institute of Bioinformatics and Structural Biology, National Tsing Hua University, Hsinchu, Taiwan; 6 Department of Biological Science and Technology, National Yang Ming Chiao Tung University, Hsinchu, Taiwan; 7 Program in Environmental and Occupational Medicine, Kaohsiung Medical University, Kaohsiung, Taiwan

**Keywords:** hepatocellular carcinoma, metabolic dysfunction–associated steatotic liver disease (MASLD), zebrafish, macrophages, phenotypic response surface-guided optimization (PRS-OPT)

## Abstract

**Objectives:** Hepatocellular carcinoma (HCC) arising in metabolic dysfunction–associated steatotic liver disease (MASLD) develops under lipid-rich stress and inflammatory remodeling, which can alter therapeutic windows. We aimed to determine whether phenotypic response surface–guided optimization (PRS-OPT) can nominate hepatocyte-sparing propolis–metformin–regorafenib (PMR) dose windows that retain antitumor activity under MASLD-like fatty-acid (FA) stress and translate to an *in vivo* immune endpoint. **Methods:** PMR combinations were profiled in hepatoma cell lines (PLC/PRF/5 and HepG2) and non-malignant hepatocytes (THLE-2) under FA-free and FA-enriched conditions. Quadratic response surfaces were fitted and used for constrained dose nomination, followed by *in vitro* validation. Cell-death contributions were assessed by inhibitor-rescue, Annexin V/PI flow cytometry, and immunoblotting. PRS-OPT was extended to zebrafish MASLD-HCC to optimize dosing against hepatic macrophage accumulation, with longitudinal imaging and qPCR endpoints. **Results:** FA exposure shifted the feasible efficacy–tolerability landscape, leading PRS-OPT to nominate a distinct FA-optimized PMR window with improved hepatocyte preservation while maintaining tumor suppression. Validation confirmed tumor-preferential activity across hepatoma lines with hepatocyte sparing. Mechanistic assays supported apoptosis with context dependence and evidence consistent with a ferroptosis-related component in HepG2. In zebrafish MASLD-HCC, PRS-OPT–nominated dosing reduced hepatic macrophage accumulation and improved disease-relevant transcriptional and morphological readouts. **Conclusions:** PRS-OPT enables interpretable, multi-objective dose nomination for MASLD-relevant HCC contexts, and establishes PRS-OPT as an interpretable, multi-objective framework for regimen nomination that is directly extensible to additional phenotypic endpoints for preclinical evaluation.

## 1 Introduction

Hepatocellular carcinoma (HCC) remains a leading cause of cancer mortality worldwide [1]. Its etiologic landscape is changing as metabolic dysfunction–associated steatotic liver disease (MASLD; formerly non-alcoholic fatty liver disease) and its inflammatory subtype, metabolic dysfunction–associated steatohepatitis (MASH), become increasingly prevalent [2]. MASLD/MASH is characterized by steatosis, hepatocellular injury, and fibrosis [3], and can promote hepatocarcinogenesis even without cirrhosis [4]. Accumulating evidence further indicates that MASLD-associated HCC differs from viral or alcohol-related disease at molecular and immunologic levels, including altered metabolism, stromal remodeling, and innate immune activation [5]. These differences limit direct extrapolation from viral hepatitis–enriched evidence bases and motivate etiologically-informed therapeutic strategies.

Although tyrosine kinase inhibitors and immune checkpoint inhibitors (ICIs) have expanded options for advanced HCC, most pivotal trials were enriched for viral hepatitis–related disease [6]. Clinical and translational studies suggest that MASLD-associated HCC can exhibit features associated with reduced immunotherapy benefit, including impaired cytotoxic T-cell infiltration and function [7], and a lower tumor mutational burden [8], with additional evidence supporting etiologic differences in immune responsiveness [9]. Together, these observations highlight a central challenge: regimens optimized in virally driven HCC may not perform similarly in metabolically driven tumors. Therapeutic development for MASLD-HCC therefore requires models and selection criteria that explicitly incorporate lipid-rich metabolic stress and immune context.

Prior combination-therapy optimization often relies on empirical “screen-and-score” designs (e.g., fixed-ratio or checkerboard matrices) coupled with interaction reference models such as Bliss independence or Loewe additivity to quantify synergy/antagonism and prioritize candidate combinations for follow-up [10]. While effective for interaction ranking, dose selection is frequently performed as a downstream step guided by observed efficacy windows and tolerability, rather than being posed as an explicit constrained optimization problem [11]. Complementary computational approaches—including mechanistic pathway models and machine-learning predictors—can help prioritize combinations, but often require substantial training data, may be context-dependent, and do not always yield transparent, testable dose constraints for a specific biological setting [12]. Phenotypic response-surface platforms address these gaps by using small, strategically sampled experiments to model multidrug dose–phenotype landscapes and prospectively nominate regimens for experimental validation [13,14]. A remaining gap is the prospective dose nomination under MASLD-like lipid stress while explicitly constraining hepatocyte tolerance and accommodating immune-relevant endpoints. Phenotypic response surface–guided optimization (PRS-OPT) addresses this gap by fitting an interpretable quadratic response surface (by ordinary least squares) and applying constrained multi-objective optimization to nominate dose sets that prioritize tumor suppression while preserving hepatocyte viability within the tested dose space.

Drug repurposing can accelerate therapy development by leveraging established safety and pharmacokinetic profiles [15]. To reduce trial-and-error dose selection in a combination setting, the PRS-OPT workflow was developed [16]. This data-driven framework maps multidrug dose–phenotype relationships using a small, strategically designed experimental set and supports rapid re-optimization across disease-relevant contexts by capturing nonlinear and interaction effects within a quadratic surface model [16].

The PRS-OPT workflow was applied to a three-component regimen comprising Taiwan propolis extract, metformin, and regorafenib (PMR), selected for complementary actions relevant to HCC. Metformin has been reported to potentiate kinase inhibitors [17], influence ferroptosis-associated pathways [18], modulate AMPK/JNK/IL-8 signaling [19], and reshape the tumor immune microenvironment, including effects on regulatory T cells [20], myeloid-derived suppressor cells [21], and CD8^+^ T-cell infiltration [22]. Propolis, a flavonoid- and phenolic-rich natural product, exhibits antiproliferative, pro-apoptotic, antioxidant, and anti-inflammatory activities across cancer models, with reported effects on oxidative stress, mitochondrial function, and MAPK signaling [23], and a safety profile supportive of adjuvant use [24]. Regorafenib is an oral multi-kinase inhibitor targeting VEGFR, PDGFR, FGFR, and RAF kinases [25], and is approved as a systemic therapy for patients with advanced HCC who progressed on sorafenib based on the phase III RESORCE trial [26]. This study tests the hypothesis that PRS-OPT–guided, constraint-based dose nomination can identify hepatocyte-sparing PMR dosing windows that retain antitumor activity under MASLD-like FA stress and that this framework can be extended to optimize an *in vivo* immune endpoint.

Zebrafish provide a genetically tractable *in vivo* system with optical access for real-time imaging of tumor and immune dynamics [27], and high-fat feeding recapitulates key metabolic and histologic features of MASLD [28]. Transgenic lines enable visualization and quantification of hepatic macrophages and neutrophils, and allow assessment of hepatocyte nuclear-to-cytoplasmic ratio as a morphological readout relevant to dysplasia. Prior studies have established zebrafish models that mirror stepwise human hepatocarcinogenesis [29,30] and support therapeutic evaluation with immune phenotyping [31,32,33,34]. Zebrafish have also been used to assess repurposed agents and natural products in MASLD-associated HCC–relevant contexts, including examples targeting STAT3-linked pathways [35] and metabolic-immune imbalance [36]. Collectively, these data support zebrafish as a practical bridge between *in vitro* screening and subsequent mammalian validation.

This study tests the hypothesis that phenotypic response surface–guided optimization (PRS-OPT) can nominate hepatocyte-sparing propolis–metformin–regorafenib (PMR) dosing windows that retain antitumor activity under MASLD-like FA stress and can be extended to an *in vivo* immune endpoint. To test this hypothesis, we pursued three objectives: (i) to fit PRS-OPT models under FA-free and FA-enriched conditions and nominate PMR dose windows that reduce hepatoma viability while meeting a predefined hepatocytes-sparing constraint; (ii) to characterize context- and cell line–dependent death programs using pharmacologic rescue assays together with apoptosis/ferroptosis-associated readouts; and (iii) to evaluate translational *in vivo* endpoints in zebrafish MASLD-HCC models, including hepatic macrophage accumulation, inflammatory-marker expression, and hepatocyte morphological readouts. Together, these experiments evaluate PRS-OPT as an interpretable, multi-objective strategy for context-aware regimen nomination in metabolically driven HCC and provide a foundation for subsequent mammalian validation and biomarker-guided refinement.

## 2 Materials and Methods

To test the stated hypothesis, we used an integrated workflow combining (i) *in vitro* viability profiling of *Propolis–Metformin–Regorafenib* (PMR) in hepatoma and non-malignant hepatocytes under fatty acid (FA)–free vs. FA-enriched conditions; (ii) PRS-OPT fitting and constrained dose nomination strictly within experimentally tested bounds; (iii) mechanism-oriented assays including inhibitor-rescue, Annexin V/PI flow cytometry, and immunoblotting; and (iv) zebrafish validation in MASLD-HCC and comparator HCC models using longitudinal immune imaging and hepatic molecular/morphological endpoints. Step-by-step recipes, stock preparation, instrument/acquisition settings, image-analysis parameters, and PRS-OPT implementation notes are provided in Supplementary Methods S1–S15.

### 2.1 Cell Culture

Human hepatocellular carcinoma cell lines PLC/PRF/5 (Bioresource Collection and Research Center [BCRC], 60223, Hsinchu, Taiwan) and HepG2 (BCRC, 60025), immortalized human hepatocyte THLE-2 (American Type Culture Collection [ATCC], CRL-2706, Manassas, VA, USA) and the THP-1 monocytes (ATCC TIB-202) were used. PLC/PRF/5 and HepG2 cells were maintained in Dulbecco’s modified Eagle medium (DMEM, Gibco, 12100046, Waltham, MA, USA) supplemented with 10% fetal bovine serum (FBS, Gibco, 11965-092) and 1% penicillin–streptomycin (P/S, Gibco, 15140-122) at 37°C, 5% CO_2_. THLE-2 cells were cultured in BEGM^®^ BulletKit (Lonza, CC-3170, Walkersville, MD, USA) prepared without gentamicin/amphotericin B and epinephrine, supplemented with epidermal growth factor (EGF, Merck Millipore, 01-107/01-407, Burlington, Massachusetts, USA, final 5 ng/mL) and phosphoethanolamine (Sigma-Aldrich, P0503, St. Louis, MO, USA, final 70 ng/mL), 10% FBS, and 1% P/S. THP-1 cells were maintained in RPMI-1640 (Gibco, 31800022) with 10% FBS and 1% P/S, differentiated to macrophage-like M0 with Phorbol 12-myristate 13-acetate (PMA, Sigma-Aldrich, 79346, final 100 nM), then polarized to M1 with IFN-γ (Interferon-gamma, R&D Systems, 285-IF, Minneapolis, MN, USA, final 2 ng/mL) + LPS (Lipopolysaccharides, Sigma-Aldrich, L2630, final 100 ng/mL), or to M2 with IL-4 (Interleukin-4, Sigma-Aldrich, I4269, final 20 ng/mL). Cell authentication, mycoplasma testing and routine culture practices are detailed in Supplementary Methods S1.

### 2.2 Taiwan Propolis: Source, Extraction, and Handling

Raw Taiwan propolis (*Apis mellifera*; Taiwan origin) was obtained as a research-grade material from GoldWise Co., Ltd. (Taipei, Taiwan). Propolis was extracted in 75% (v/v) ethanol at 200 mg raw material/mL solvent and clarified by 0.22 μm sterile-filtration to generate a stock reported as raw-material equivalents. Working dilutions were freshly prepared in culture medium; with ethanol vehicle-matched across groups and maintained at ≤0.1% (v/v). Expanded extraction workflow, storage/handling, unit conversion, and batch traceability are provided in Supplementary Methods S2.

### 2.3 Fatty Acid (FA) Preparation and Treatment

To model MASLD-like lipotoxic stress, oleic acid (OA; Sigma-Aldrich, O1008) and palmitic acid (PA; Sigma, P0500) were prepared as BSA-conjugated stocks using fatty acid–free BSA (Sigma-Aldrich, A8806) and added to culture medium at final concentrations of 0.5 mM OA + 0.25 mM PA. FA exposure was applied concurrently with drug treatment. Vehicle controls were matched for fatty acid–free BSA and ethanol (≤0.1% (v/v)). Stock preparation and vehicle-matching details are described in Supplementary Methods S3.

### 2.4 Cell Viability (CCK-8) Assay

PLC/PRF/5, HepG2 (BCRC, 60025), THLE-2, and M1 or M2 cells were seeded in 96-well plates (3000 cells/well) and treated with propolis, metformin (MedChemExpress, HY-17471A, Monmouth Junction, NJ, USA), and regorafenib (MedChemExpress, HY-10331) single agents or combinations, with or without FA for the indicated durations (as specified in figure legends). Viability was quantified using CCK-8 (Dojindo, CK04, Kumamoto, Japan, 10 μL per well) and absorbance at 450 nm measured on a microplate reader (Infinite^®^ M200 PRO, Tecan Group Ltd., Männedorf, Switzerland). Plate layout, background correction, and normalization are provided in Supplementary Methods S4.

### 2.5 PRS-OPT Dose Optimization and Constrained Nomination

Phenotypic Response Surface–Optimization (PRS-OPT) is a constrained response-surface modeling and optimization workflow used to fit multidrug dose–response surfaces and nominate dose sets within the experimentally tested dose bounds. For *in vitro* PMR profiling, each dose condition was measured in triplicate wells per plate (technical replicates), and the full experiment was repeated in three independent runs performed on different days using independently prepared cell cultures (biological replicates), unless otherwise stated. Background-corrected and normalized CCK-8 readouts were first averaged across technical replicates to generate a single value per condition for each biological replicate; replicate-level responses were then aggregated to yield mean viability inputs for PRS-OPT model fitting. Dose nomination aimed to maximize tumor suppression while preserving non-malignant hepatocyte viability, subject to feasibility constraints defined by the tested dose space.

For *in vivo* optimization, zebrafish were treated and quantified across independent experimental batches (biological replicates: *n* = 2–3 independent batches; ~10–20 fish/group/batch). For each fish, imaging-derived hepatic macrophage infiltration metrics were calculated as macrophage density ratios at 15 dpf relative to the corresponding baseline time point (9 dpf or 12 dpf, depending on the experiment). Fish-level ratios were used as outputs for PRS-OPT modeling and constrained dose nomination. Nominated PMR doses were selected to minimize macrophage accumulation while remaining within the experimentally tested dose bounds.

**Workflow overview.** PRS-OPT proceeded in five steps: (i) define model inputs and outputs (drug concentrations as inputs; viability or macrophage-density ratios as outputs), (ii) fit an interpretable quadratic response-surface model capturing main effects and drug–drug interactions, (iii) assess model adequacy using prespecified diagnostics and robustness checks, (iv) perform constrained optimization within the experimentally tested bounds to nominate dose sets that satisfy explicit biological constraints, and (v) prospectively validate nominated dose sets in independent *in vitro* experiments and zebrafish assays. The mathematical formulation, diagnostics, solver settings, and implementation details are provided in Supplementary Methods S5.

**Model adequacy and robustness checks.** Model adequacy was assessed using goodness-of-fit metrics and prespecified diagnostics. Fit was summarized by predicted-versus-observed plots and the coefficient of determination (R^2^). Residual-based diagnostics were performed to evaluate key OLS assumptions, including normality (Shapiro–Wilk test and Q–Q plots), homoscedasticity (Breusch–Pagan test and residuals-versus-fitted plots), and error independence (Durbin–Watson statistic and residuals-versus-run-order plots). Multicollinearity among polynomial terms was assessed by variance inflation factors (VIFs) on the mean-centered design matrix. Predictive robustness and uncertainty were evaluated using K-fold cross-validation (CV RMSE) and nonparametric bootstrap resampling. Diagnostic plots and summary statistics are provided in the Supplementary Information, with full technical details in Supplementary Methods S5 and S6.

### 2.6 Predictor Scaling, Collinearity, and Model Diagnostics

Because the three agents span different numerical concentration ranges, models were fit using concentrations in their original units within the experimentally tested bounds, and z-score standardization was performed as a sensitivity analysis; standardization did not materially change model fit metrics or the locations of model-nominated optima (Supplementary Information). Multicollinearity among main effects and polynomial terms was assessed using variance inflation factors (VIFs) on the mean-centered design matrix; we prespecified VIF ≥ 3 as indicating potentially severe multicollinearity, and all terms exhibited VIF < 3 (Supplementary Information), indicating no evidence of severe multicollinearity.

Key OLS assumptions were evaluated using residual-based diagnostics, including normality (Shapiro–Wilk), heteroscedasticity (Breusch–Pagan), and error independence (Durbin–Watson), together with diagnostic plots shown in the Supplementary Information: Q–Q plots and residuals histograms (SI1), residuals versus fitted values (SI2), and residuals versus experiment/run order (SI3). Where deviations from ideal assumptions were observed in specific conditions, OLS was retained for interpretability, and dose nomination was supported by out-of-sample performance and uncertainty estimation using K-fold cross-validation and nonparametric bootstrap. All primary diagnostics and robustness evaluations are summarized in the Supplementary Figs. S10–S12, with full technical details and implementation parameters provided in Supplementary Methods S6.

### 2.7 Oil Red O Staining and Quantification

Oil Red O staining was performed to evaluate intracellular lipid accumulation after 72 h under four defined conditions: (1) vehicle control (BSA/ethanol-matched), (2) FA only (0.5 mM oleic acid + 0.25 mM palmitic acid), (3) PMR only (propolis–metformin–regorafenib at the indicated doses), and (4) FA + PMR. Cells were fixed with 10% formalin at room temperature and stained with Oil Red O (Sigma-Aldrich, O-0605H) for 10 min, washed to remove unbound dye, and imaged. For quantification, the retained dye was eluted with isopropanol and absorbance was measured at 500 nm. Absorbance values were blank-subtracted and normalized to the corresponding vehicle control level (reported as fold-change), to ensure quantitative comparability across experiments. Full procedures and calculation details are provided in Supplementary Methods S7.

### 2.8 Inhibitor Pretreatment for Cell Death Pathway Interrogation

To probe PMR-induced death mechanisms, cells (3000 cells/well; 96-well plates) were treated with PMR in the presence or absence of pathway modulators: N-acetylcysteine amide (NAC, 1 mM; TargetMol Chemicals Inc., T5518, Wellesley Hills, MA, USA), Z-VAD-FMK (50 μM; TargetMol, T6013), ferrostatin-1 (10 μM; TargetMol, T6500), necrostatin-1 (50 μM; TargetMol, T1847), and 3-methyladenine (10 μM; TargetMol, T1879). Modulators were applied as pretreatments for 4 h, followed by co-treatment with PMR plus the same modulator for 24 h prior to CCK-8 readout; stock preparation, solvents, and vehicle controls are provided in Supplementary Methods S8.

### 2.9 Annexin V-FITC Staining and Flow Cytometry

Cells were seeded in 6-well plates (2.5 × 10^5^ cells/well). Apoptosis was quantified by Annexin V-FITC/PI staining using an apoptosis detection kit (Abcam ab14085, Cambridge, UK) after 48 h treatment under defined conditions: (1) vehicle control (BSA/ethanol-matched), (2) PMR alone (propolis–metformin–regorafenib at the indicated doses), (3) FA alone (0.5 mM oleic acid and 0.25 mM palmitic acid), and (4) PMR and FA. Cell populations were defined as viable (Annexin V^−^/PI^−^), early apoptotic (Annexin V^+^/PI^−^), late apoptotic (Annexin V^+^/PI^+^), and necrotic (Annexin V^−^/PI^+^). Samples were analyzed on an Attune™ NxT flow cytometer (Thermo Fisher Scientific, Waltham, MA, USA), acquiring ≥10,000 events per sample after exclusion of debris, and data were analyzed using FlowJo™ (BD, Ashland, OR, USA). Detailed staining volumes, acquisition settings, and gating strategy are provided in Supplementary Methods S9.

### 2.10 Western Blotting

Protein expression changes after drug treatment were assessed by immunoblotting. Cells were treated with vehicle control, PMR-only, FA-only, or PMR + FA for 0, 24, or 48 h, washed with cold PBS, and lysed in RIPA buffer (150 mM NaCl, 5 mM EDTA, 25 mM Tris-HCl pH 7.6, 1% NP-40, 1% sodium deoxycholate, 0.1% SDS) supplemented with a protease inhibitor cocktail (MedChemExpress, HY-K0010) and phosphatase inhibitor cocktails (MedChemExpress, HY-K0021, HY-K0022, HY-K0023). Protein concentration was determined by BCA assay (Thermo Fisher Scientific Pierce™, 23227), and equal amounts of total protein (30 μg/lane) were separated on Bis-Tris precast gels (MOEKO BIO, P20412-010, Taipei, Taiwan) using a Bio-Rad Mini-PROTEAN^®^ electrophoresis system (Bio-Rad Laboratories, Hercules, CA, USA), then transferred to PVDF membranes (0.22 μm) (Pall, BSP0161). 

Membranes were blocked in 3% BSA/TBST (TBST: Protech Technology Enterprise, BF204, Taipei, Taiwan) for 1 h at room temperature, incubated with primary antibodies overnight at 4°C, washed in TBST (3 × 5 min), and incubated with HRP-conjugated secondary antibodies for 1 h at room temperature. Primary antibodies and working dilutions were: β-actin (Proteintech, 20536-1-AP, 1:5000), caspase-3 (GeneTex, GTX110543, Rosemont, IL, USA, 1:1000), cleaved caspase-3 (Asp175) (Cell Signaling Technology, 9661, Danvers, MA, USA; 1:1000), PARP (Cell Signaling Technology, 9542, Danvers, MA, USA; 1:1000), and Bax (Cell Signaling Technology, 2772, 1:1000). Secondary antibodies were goat anti-mouse IgG-HRP (GeneTex, GTX213111-01, Irvine, CA, USA; 1:5000) and anti-rabbit IgG-HRP (Cell Signaling Technology, 7074, 1:5000). Signals were developed using T-Pro LumiLong Plus chemiluminescent Substrate (T-Pro Biotechnology, JT96-K004M, New Taipei City, Taiwan) and captured on a ChemiDoc™ MP Imaging System (Bio-Rad Laboratories, Hercules, CA, USA). Band intensities were quantified using Fiji/ImageJ (v2.16.0; National Institutes of Health [NIH], Bethesda, MD, USA), normalized to β-actin, and expressed relative to the 0 h control within each condition. Additional technical details are provided in Supplementary Methods S10.

### 2.11 Zebrafish Ethics, Husbandry, Experimental Animals, and Study Design

All zebrafish experiments were approved by the NHRI Institutional Animal Care and Use Committee (NHRI-IACUC-113155-A) and performed in the Taiwan Zebrafish Core Facility (NHRI), an AAALAC-accredited facility. Zebrafish (*Danio rerio*) were maintained at 28°C under a 14:10 h light:dark cycle using standard facility husbandry. Experiments used embryos/larvae (3 h post-fertilization [hpf] to 15 days post-fertilization [dpf]); sex was not determined at these larval stages. The transgenic lines used enabled visualization of macrophages (*mpeg:mCherry*), neutrophils (*mpx:EGFP*), hepatocyte membranes (*fabp10a:Palmitoyl-mTurquoise*), nuclei (*fabp10a:H2A-mCherry*), and myocardium (*myl7:EGFP*). 

**Study design and experimental unit.** The experimental unit was an individual embryo/larva. For each assay, larvae were allocated to predefined experimental groups (see Section 2.12 and Section 2.13) and quantified using prespecified endpoints (survival/malformations for embryotoxicity; diet/drug-dependent immune infiltration and hepatic molecular/morphological endpoints for disease models). Expanded husbandry, crossing schemes, complete genotype definitions, and line validation are provided in Supplementary Methods S11.

### 2.12 Embryotoxicity Assay

Embryotoxicity of Taiwan propolis was assessed from 3 hpf to 5 dpf using a daily renewal exposure paradigm. After baseline screening to remove non-viable embryos, embryos were allocated to vehicle control or propolis treatment at 10^−^^6^, 10^−^^5^, 10^−^^4^, or 10^−^^3^ (reported as fold-dilutions of the propolis stock) with daily solution renewal. The experimental unit was an individual embryo. Endpoints were prespecified as survival (recorded daily) and gross developmental abnormalities, including pericardial edema and tail malformation, assessed at 5 dpf by stereomicroscopy. Sample size and replication were *n* = 10 embryos per condition, repeated across 3 independent clutches/experiments. Embryos that were non-viable at baseline were excluded prior to allocation; deaths during the exposure window were included in survival analyses and excluded from malformation scoring. Detailed solution preparation, renewal procedures, scoring criteria, and blinding/randomization implementation are provided in Supplementary Methods S12.

### 2.13 Larval Diets, Drug Treatment, Randomization, and Blinding, and Welfare Considerations

For MASLD-HCC experiments, larvae were assigned at 5 dpf to either a high-fat diet (24% fat) or control diet (12% fat). Pigmentation was suppressed for imaging using PTU (0.002% from 2 dpf; 0.003% from 9 dpf). Drug treatment was performed by daily bath exposure to PMR in 24-well plates (one larva per well) from 10–15 dpf with daily renewal of treatment solution. Larvae were randomly allocated to experimental groups, and images were coded prior to quantification so that image analyses were performed blinded to group identity. Larvae were monitored daily for overt morbidity during husbandry and treatment; anesthesia and handling for imaging followed IACUC-approved procedures (details in Supplementary Methods S13 and imaging methods). Sample sizes for each experimental group and the number of independent experimental batches are reported in the relevant figure legends and Supplementary Methods. Statistical methods are described in Section 2.16.

### 2.14 Confocal Microscopy

Larvae were anesthetized with tricaine (MS-222; Sigma-Aldrich/Merck, St. Louis, MO, USA) and mounted in 1.25% low-melting agarose (Zymeset, BAG102, Taipei, Taiwan) on glass-bottom dishes for imaging. Confocal imaging was performed at defined time points (e.g., 9, 12, and 15 dpf, as indicated) using a Leica TCS SP5 (Leica Microsystems, Wetzlar, Germany) or Leica Stellaris 8 confocal microscope (Leica Microsystems), with identical acquisition settings maintained within each experiment for quantitative comparisons. Hepatic macrophage (mpeg:mCherry) and neutrophil (mpx:EGFP) signals were quantified within a predefined liver region of interest (ROI), and densities were normalized to liver area to account for inter-individual liver-size variability. Immune “infiltration ratios” were calculated as the area-normalized density at 15 dpf divided by that at 9 dpf (or 12 dpf, as indicated). ROI definition, ImageJ-based analysis workflow, normalization, and thresholding parameters are provided in Supplementary Methods S14.

### 2.15 RNA Extraction, cDNA Synthesis, and qPCR

Total RNA was isolated from individual zebrafish larvae using a column-based kit (NucleoSpin^®^ RNA, MACHEREY-NAGEL GmbH & Co. KG, 740955.50, Düren, Germany), reverse-transcribed to cDNA (iScript™, Bio-Rad, 1708890), and analyzed by SYBR Green qPCR (Thermo Fisher Scientific, 4385618) on a QuantStudio™ 5 Real-Time PCR system (Applied Biosystems/Thermo Fisher Scientific, Waltham, MA, USA). Relative expression was calculated using an efficiency-corrected ΔΔCt method with actin as the reference; based on assay calibration, amplification efficiency was 1.94 per cycle (≈94%), and fold change was computed as 1.94^−ΔΔCt^. Primer sequences are listed in Table S1. Expanded homogenization, RNA QC, reaction setup, plate design, and efficiency calibration details are provided in Supplementary Methods S15.

### 2.16 Statistical Analysis

Statistical analyses were performed in GraphPad Prism (v10, GraphPad Software, San Diego, CA, USA). For comparisons among multiple groups, one-way ANOVA with Tukey’s post hoc test was used. When two independent variables were analyzed, two-way ANOVA with Bonferroni’s correction was applied. Data are presented as mean ± SD, and *p* < 0.05 was considered statistically significant (*0.01 < *p* ≤ 0.05; **0.001 < *p* ≤ 0.01; ***0.0001 < *p* ≤ 0.001; ****p ≤ 0.0001). Where applicable, the specific statistical test and sample size (*n*) for each experiment are indicated in the corresponding figure legends.

## 3 Results

### 3.1 Source Selection, Single-Agent Profiling, and Cell-Model Rationale

To benchmark propolis sources, PLC/PRF/5 and HepG2 hepatoma cells were profiled with graded propolis for 72 h; IC_50_ values indicated that the Taiwan propolis was most potent (Fig. S1). For clarity, propolis concentrations are expressed as dilutions of a 200 mg/mL stock (raw-equivalent) and can be converted to mass concentration using the relationship above (e.g., 1 × 10^−^^4^ = 20 μg/mL). IC_50_ values for metformin and regorafenib in both lines (Fig. S2) defined concentration ranges for combination testing.

To capture etiologic and genetic heterogeneity relevant to MASLD-HCC, two canonical HCC models and a non-malignant control were used: PLC/PRF/5 (adult HCC, integrated HBV DNA, secretes HBsAg, TP53 R249S) and HepG2 (HBV-negative, TP53 wild type, stabilizing CTNNB1/β-catenin exon-3 alteration), alongside THLE-2 normal hepatocytes. This panel enables tumor selectivity assessment across divergent backgrounds.

### 3.2 PMR Reduces Hepatoma Viability while Sparing THLE-2 Cells and Remains Active under Fatty Acid Exposure

**Figure 1:**
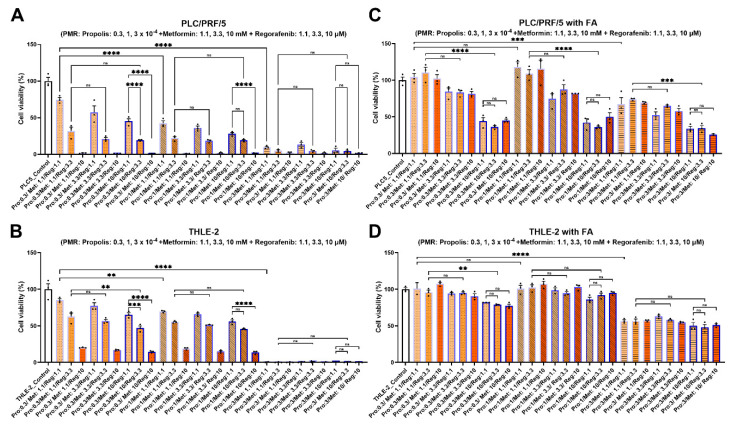
**Effects of PMR combinations on hepatoma and hepatocyte viability with and without fatty acid enrichment.** PLC/PRF/5 and THLE-2 cells were treated with indicated combinations of propolis, metformin, and regorafenib for 72 h under fatty acid (FA)-free or FA-enriched conditions (**A**) PLC/PRF/5, FA-free. (**B**) THLE-2, FA-free. (**C**) PLC/PRF/5, FA-enriched. (**D**) THLE-2, FA-enriched. Propolis was dosed as fold-dilutions of a 200 mg/mL 75% ethanol extract stock (raw-material equivalents); for reference, 1 × 10^−^^4^ corresponds to 20 μg/mL and 0.3 × 10^−^^4^ corresponds to 6 μg/mL (raw-equivalent). Vehicle ethanol was matched across conditions and maintained at ≤0.1% (v/v). Data are mean ± SD from three independent experiments. Statistical significance notation follows Methods (**p < 0.01; ***p < 0.001; ****p < 0.0001; ns, not significant). PMR: propolis-metformin-regorafenib.

Guided by the single-agent activity windows, we evaluated *Propolis-Metformin-Regorafenib* (PMR) in PLC/PRF/5 and THLE-2 cells under fatty acid (FA)-free conditions and during concurrent FA exposure (0.5 mM oleic acid + 0.25 mM palmitic acid, BSA-conjugated; vehicle-matched controls). Under FA-free conditions, PMR decreased PLC/PRF/5 viability in a dose-dependent manner while preserving comparatively higher THLE-2 viability across the active dose range (Fig. 1A,B). With FA co-treatment, PMR-induced cytotoxicity in PLC/PRF/5 was partially attenuated, whereas THLE-2 viability remained high (Fig. 1C,D), indicating a context-dependent shift in drug response under lipid-enriched conditions.

**Figure 2:**
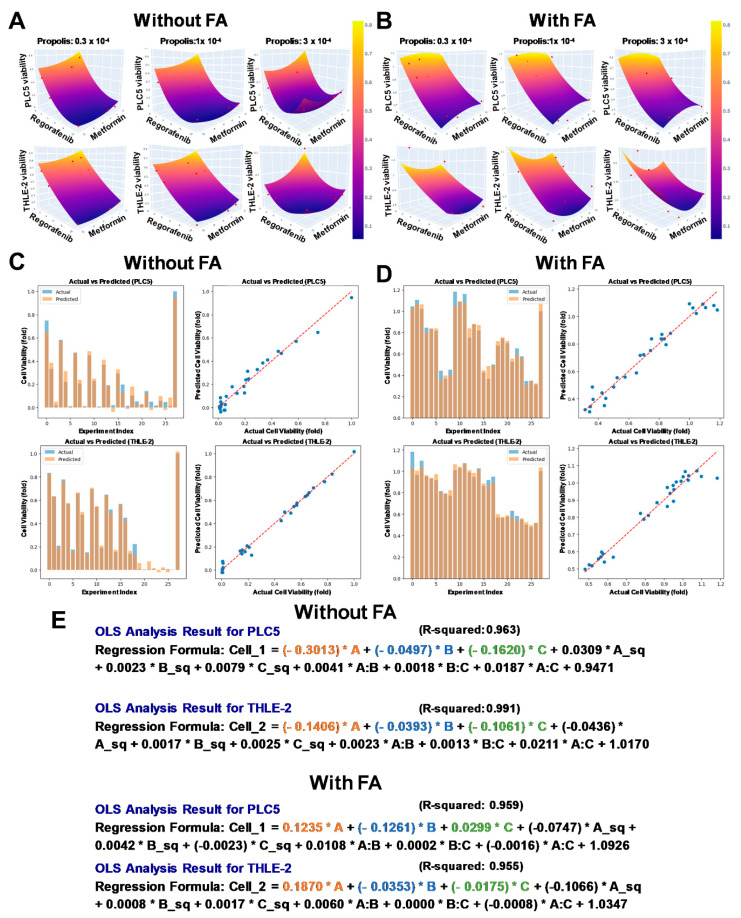
**Predictive modeling of combinatorial effects on cell viability with and without fatty acid exposure.** (**A**,**B**) PRS-OPT generated response surface plots illustrating the effects of propolis, metformin, and regorafenib combinations on cell viability in PLC/PRF/5 and THLE-2 cells, under fatty acid-free (**A**) and fatty acid-enriched (**B**) conditions. Red dots represent experimental input data used for model fitting. Propolis concentrations are expressed as fold-dilutions of a 200 mg/mL ethanol extract stock (raw-material equivalents) and are convertible to approximate working concentrations (e.g., 1 × 10^−^^4^ = 20 μg/mL; 0.3 × 10^−^^4^ = 6 μg/mL, raw-equivalent). The ethanol vehicle was matched across conditions and maintained at ≤0.1% (v/v). (**C**,**D**) Predicted versus actual viability plots show concordance between model estimates and measured data. (**E**) Ordinary Least Squares (OLS) regression coefficients summarize the directionality and relative magnitude of component contributions (propolis, metformin, regorafenib) in both cell lines, with and without fatty acid exposure.

To nominate hepatocyte-sparing regimens, quadratic phenotypic response surfaces were fit, and the PRS-OPT workflow was applied using constrained multi-objective optimization to the PMR dose matrix. The fitted surfaces captured nonlinear dose effects and interaction structure across the tested concentration space for both PLC/PRF/5 and THLE-2 (Fig. 2A,B). Predicted-versus-observed viabilities plots showed close concordance (Fig. 2C,D), supporting model adequacy for dose nomination. Coefficient patterns suggested condition-dependent shifts in component contributions (Fig. 2E): in FA-free media, propolis contributed more strongly to PLC/PRF/5 inhibition than to THLE-2 suppression, whereas under FA, propolis effects shifted toward a more viability-preserving profile, particularly in THLE-2. Metformin exhibited modest effects in FA-free conditions but a larger inhibitory contribution in PLC/PRF/5 under FA with limited impact on THLE-2, consistent with enhanced tumor-selective activity in lipid-enriched states.

**Figure 3:**
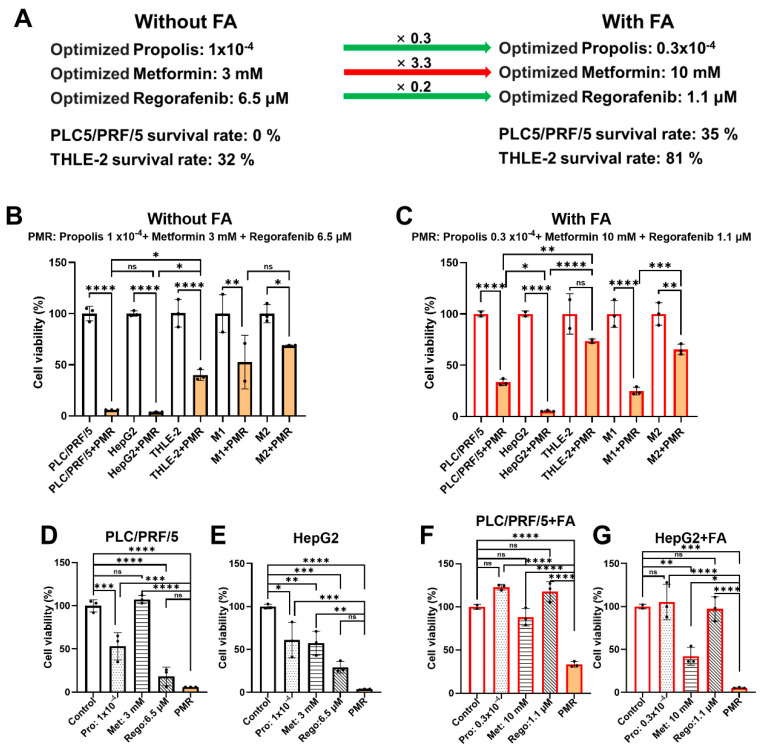
**Experimental validation of PRS-OPT–nominated PMR windows with and without fatty acid enrichment.** (**A**) PRS-OPT nominates distinct PMR dose sets under FA-free and FA-enriched conditions. Under FA-free conditions, the nominated regimen (propolis 1 × 10^−^^4^, metformin 3 mM, regorafenib 6.5 μM) maximized tumor suppression but reduced THLE-2 viability; under FA, the nominated regimen (propolis 0.3 × 10^−^^4^, metformin 10 mM, regorafenib 1.1 μM) improved hepatocyte preservation while maintaining tumor inhibition (values shown in panel). Propolis is expressed as fold-dilutions of a 200 mg/mL extract stock (raw-material equivalents; 1 × 10^−^^4^ ≈ 20 μg/mL; 0.3 × 10^−^^4^ ≈ 6 μg/mL). (**B**,**C**) Validation of the nominated PMR regimens across PLC/PRF/5, HepG2, THLE-2, and polarized macrophages (M1, M2) under FA-free (**B**) and FA-enriched (**C**) conditions. (**D**–**G**) Component dissection comparing monotherapies versus the full PMR combination in PLC/PRF/5 and HepG2 under FA-free (**D**,**E**) and FA-enriched (**F**,**G**) conditions. Data are mean ± SD from independent experiments; statistical testing and significance notation follow Methods (*p < 0.05; **p < 0.01; ***p < 0.001; ****p < 0.0001; ns, not significant).

PRS-OPT nominated an FA-free optimum of propolis 1 × 10^−^^4^ of the 200 mg/mL stock (≈20 μg/mL raw-equivalent), metformin 3 mM, and regorafenib 6.5 μM (Fig. 3A), yielding near-complete suppression of PLC/PRF/5 viability with ~32% residual THLE-2 viability. Under FA exposure, the nominated regimen reduced propolis to 0.3 × 10^−^^4^ (≈6 μg/mL raw-equivalent) and regorafenib to 1.1 μM while increasing metformin to 10 mM, predicting ~35% residual PLC/PRF/5 viability with ~81% THLE-2 viability. This marked FA-dependent shift—most notably metformin 3 mM → 10 mM—reflects that FA exposure reshaped the fitted response surfaces and the feasibility region imposed by the hepatocyte-viability constraint, moving the model-based optimum within the same experimentally bounded dose space. Orthogonal validation in PLC/PRF/5, HepG2, and THLE-2 confirmed tumor-preferential viability reduction with hepatocyte sparing at these doses and showed additional viability inhibition in both M1- and M2-polarized macrophages (Fig. 3B,C). Component dissection indicated that single agents produced modest-to-moderate effects, whereas the full PMR combination produced the largest reductions in PLC/PRF/5 and HepG2 viability under both FA-free and FA conditions, with the clearest separation under FA (Fig. 3D–G).

### 3.3 PMR Primarily Affects Viability Rather than Uniformly Lowering Lipid Stores

Oil Red O staining showed that FA loading increased intracellular lipids in PLC/PRF/5 and HepG2, whereas THLE-2 exhibited minimal change, consistent with cell-type differences in FA handling (Fig. S3). PMR did not reduce lipid accumulation in PLC/PRF/5 and showed a trend toward increased lipid signal under FA, whereas metformin alone (and within PMR) reduced lipid signal in HepG2. These results suggest that PMR’s dominant effect in this setting is cytotoxic/antiproliferative rather than uniform lipid-lowering, with lipid reduction more evident in HepG2 than in PLC/PRF/5 (Fig. S3).

### 3.4 Multiple Cell-Death Programs Contribute to PMR Cytotoxicity, with Evidence Consistent with a Ferroptosis Bias Component in HepG2

**Figure 4:**
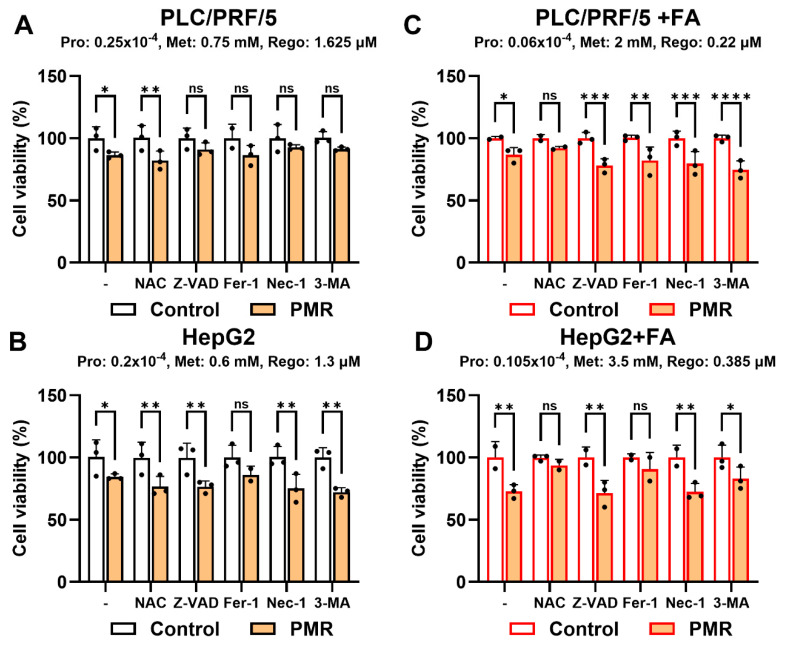
**Pharmacologic inhibitor profiling supports multiple cell-death with a ferroptosis-biased component in HepG2.** PLC/PRF/5 and HepG2 cells were treated with PMR at the indicated doses (panel headers) under fatty acid (FA)-free or FA-supplemented conditions, with pathway inhibitors applied as a pretreatment. Inhibitors were N-acetylcysteine (NAC, 1 mM), Z-VAD-FMK (50 μM), ferrostatin-1 (Fer-1, 10 μM), necrostatin-1 (Nec-1, 50 μM), and 3-methyladenine (3-MA, 10 μM). (**A**) PLC/PRF/5, FA-free. (**B**) HepG2 FA-free. (**C**) PLC/PRF/5, FA-supplemented. (**D**) HepG2, FA-supplemented. Partial rescue across inhibitors suggests engagement of multiple death programs; HepG2 rescue was most consistent with Fer-1, supporting a ferroptosis-related component. Data are mean ± SD; statistical testing and significance notation follow Methods (*p < 0.05; **p < 0.01; ***p < 0.001; ****p < 0.0001; ns, not significant).

Pathway interrogation using inhibitors of apoptosis (Z-VAD-FMK), ferroptosis (ferrostatin-1), necroptosis (necrostatin-1), autophagy (3-methyladenine), and oxidative stress (N-acetylcysteine) suggested that PMR engaged multiple death programs in PLC/PRF/5, as several inhibitors partially rescued viability under FA-free conditions (Fig. 4A). In HepG2, ferrostatin-1 was the only inhibitor that produced a significant rescue effect, supporting a prominent ferroptosis-related contribution to PMR cytotoxicity in this line (Fig. 4B). Under FA-enriched conditions, N-acetylcysteine partially restored viability in both lines (Fig. 4C,D), consistent with oxidative stress acting as a key mediator in lipid-loaded states.

### 3.5 PMR Induces Apoptosis with Cell-Type and Metabolic-Context Dependence

**Figure 5:**
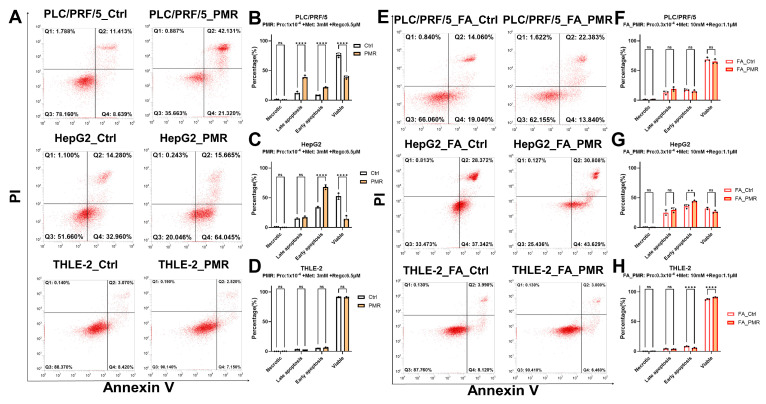
**PMR increases Annexin V–positive apoptotic fractions in hepatoma cells with limited effects in THLE-2.** Apoptosis was assessed by Annexin V–FITC/propidium iodide (PI) flow cytometry after 48 h treatment with vehicle control (Ctrl) or PMR at the indicated doses (panel headers) in PLC/PRF/5, HepG2, and THLE-2 cells under fatty acid (FA)–free or FA-supplemented conditions. (**A**) Representative dot plots showing quadrant gating for viable (Annexin V^−^/PI^−^), early apoptotic (Annexin V^+^/PI^−^), late apoptotic (Annexin V^+^/PI^+^), and PI-only necrotic (Annexin V^−^/PI^+^) populations under FA-free conditions. (**B**–**D**) Quantitation of gated populations under FA-free conditions for PLC/PRF/5 (**B**), HepG2 (**C**), and THLE-2 (**D**). (**E**) Representative dot plots under FA-supplemented conditions. (**F**–**H**) Quantification under FA-supplemented conditions for PLC/PRF/5 (**F**), HepG2 (**G**), and THLE-2 (**H**). PMR increased Annexin V–positive fractions in hepatoma cells under FA-free conditions and increased the early apoptotic fraction in HepG2 under FA, while THLE-2 showed comparatively small changes. Data are mean ± SD; statistical testing and significance notation follow Methods (**p < 0.01; ****p < 0.0001; ns, not significant).

Annexin V/PI cytometry confirmed apoptosis in PLC/PRF/5 (increases in early and late apoptotic fractions) and predominantly early apoptosis in HepG2; THLE-2 remained largely unaffected, supporting tumor selectivity (Fig. 5).

**Figure 6:**
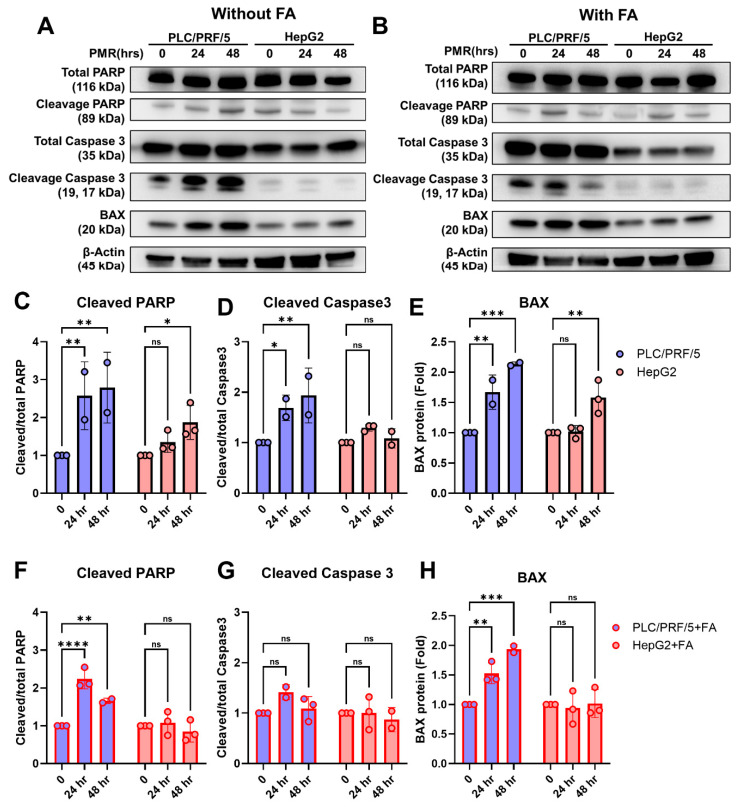
**Time-dependent induction of apoptosis-associated markers by PMR in hepatoma cells, with modulation by fatty acid loading.** (**A**,**B**) Representative immunoblots of total and cleaved PARP, total and cleaved caspase-3, and BAX in PLC/PRF/5 and HepG2 cells treated with PMR for 0, 24, or 48 h under FA-free conditions (**A**) or fatty acid (FA) supplementation (**B**). β-Actin served as a loading control. (**C**–**E**) Densitometric quantification under FA-free conditions: (**C**) cleaved/total PARP, (**D**) cleaved/total caspase-3, and (**E**) BAX abundance (normalized to β-actin). (**F**–**H**) Corresponding quantification under FA-supplementation: (**F**) cleaved/total PARP, (**G**) cleaved/total caspase-3, and (**H**) BAX abundance (normalized to β-actin). All values are shown relative to the 0 condition for each cell line. Data are mean ± SD from independent experiments; statistical testing and significance notation follow Methods (*p < 0.05; **p < 0.01; ***p < 0.001; ****p < 0.0001; ns, not significant).

Immunoblotting corroborated a caspase-dependent program in PLC/PRF/5 in FA-free media with time-dependent increases in cleaved PARP and cleaved caspase-3, alongside BAX upregulation (Fig. 6A–E). HepG2 showed BAX induction without PARP/caspase-3 cleavage, aligning with early apoptosis/ferroptosis-leaning death (Fig. 6B–E). FA blunted apoptotic markers—detectable but reduced PARP cleavage and BAX in PLC/PRF/5, and minimal induction in HepG2 (Fig. 6F–H)—consistent with FA-associated attenuation of apoptotic execution.

### 3.6 Zebrafish Establishes In Vivo Efficacy, Safety Window, and Immune Modulation

The zebrafish drug testing workflow, including embryotoxicity screen and subsequent efficacy experiments, is summarized in Fig. S4. 

To define an *in vivo* safety window for Taiwan propolis, embryos were exposed to serial dilutions (10^−3^–10^−6^) from 0–5 dpf. The 10^−3^ dilution was lethal by 1 dpf (Fig. S5A), and 10^−4^ produced developmental abnormalities by 5 dpf (Fig. S5B). Dilutions of 10^−5^–10^−6^ were well tolerated and were used as upper bounds for propolis dosing in downstream zebrafish experiments.

To model MASLD-HCC, we used a *CD36;abcg1* loss-of-function background maintained on high-fat diet and, as a general HCC comparator, a hepatocyte tert-overexpression line; both develop disease features by ~15 dpf. Crossing to *Tg(mpeg:mCherry; mpx:EGFP)* enabled longitudinal confocal imaging of hepatic macrophages (*mpeg:mCherry*, red) and neutrophils (*mpx:EGFP*, green) at 9, 12, and 15 dpf. Untreated MASLD-HCC larvae showed pronounced hepatic macrophage accumulation relative to WT (Fig. S6A). Quantification of area-normalized hepatic macrophage density within a predefined liver ROI showed that PMR reduced macrophage infiltration in a dose-dependent manner, with significant decreases at medium and high doses (Fig. S6B,C). In contrast, area-normalized hepatic neutrophil density did not differ across groups and was not altered by PMR (Fig. S6D,E), indicating macrophages are the dominant innate responders in this MASLD-HCC model.

### 3.7 PRS-OPT Extends to Immune Endpoints and Nominates Macrophage-Minimizing PMR Doses

PRS-OPT was extended to an *in vivo* immune endpoint by modeling hepatic macrophage accumulation under graded PMR inputs. Specifically, the output variable was the area-normalized hepatic macrophage density ratio at 15 dpf relative to 9 dpf (15/9), quantified from confocal images within a predefined liver ROI (see Methods). The fitted response surfaces captured the dose-dependent immune response and showed good agreement between predicted and observed values (Fig. S7A,B), nominating a PMR dose optimized to minimize macrophage accumulation (Fig. S7B). 

**Figure 7:**
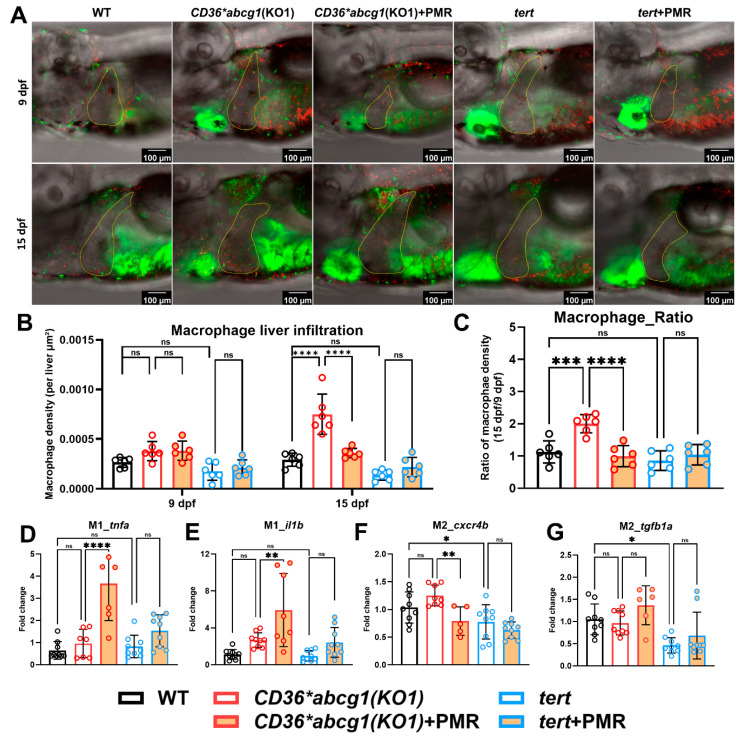
**PRS-OPT–nominated PMR dose reduces hepatic macrophage accumulation and alters macrophage marker expression in the MASLD-HCC zebrafish model.** (**A**) Representative confocal images of transgenic zebrafish liver (yellow outline) at 9 and 15 dpf in Tg(*mpeg:mCherry*; *mpx:EGFP*) WT controls, the MASLD-HCC model Tg(*mpeg:mCherry*; *mpx:EGFP*; *fabp10a:CD36*; *myl7:EGFP*); *abcg1(KO1)*, and the general HCC model Tg(*mpeg:mCherry*; *mpx:EGFP*; *fabp10a:tert*), each treated with vehicle or the PRS-OPT–nominated PMR dose (propolis 1.4 × 10^−6^, Metformin 13 μM, Regorafenib 16.5 nM). Macrophages are shown in red (*mpeg:mCherry*), neutrophils and heart are shown in green (*mpx:EGFP* and *myl7:EGFP*, respectively). Scale bars, 100 μm (shown on each micrograph). (**B**) Area-normalized hepatic macrophage density quantified at 9 and 15 dpf from a predefined liver ROI (see Methods). (**C**) Macrophage infiltration dynamics summarized as the 15 dpf/9 dpf area-normalized hepatic macrophage density ratio. (**D**–**G**) Hepatic qPCR of macrophage polarization markers, including M1-associated *tnfa* (**D**) and *il1b* (**E**) and M2-associated *cxcr4b* (F) and *tgfb1a* (**G**). Expression was processed and normalized as described in Methods and plotted relative to the indicated control group. Data are shown as mean ± SD with individual larvae overlaid; statistical comparisons are indicated on the plots (*p < 0.05; **p < 0.01; ***p < 0.001; and ****p < 0.0001; ns, not significant).

Prospective validation in two disease contexts demonstrated etiology-dependent effects. Compared with WT, MASLD-HCC larvae (*CD36;abcg1*(KO1)) exhibited elevated macrophage density, whereas the *tert*-driven HCC did not show a comparable increase. Treatment with PRS-OPT–nominated PMR regimen reduced hepatic macrophage accumulation in MASLD-HCC toward WT levels, with minimal effect in the *tert* model (Fig. 7A–C), consistent with a model-specific immune benefit. In contrast, hepatic neutrophil density remained unchanged across genotypes and treatment conditions (Fig. S8A,B), indicating that macrophages were the dominant innate responder in this setting.

To further probe immune polarization, transcript levels of macrophage-associated markers (*tnfa*, *il1b*, *tgfb1a*, *cxcr4b*) were quantified. Baseline expression of these markers did not differ significantly between MASLD-HCC and WT larvae. Following PMR treatment, MASLD-HCC larvae showed increased *tnfa* and *il1b* and decreased *cxcr4b*, with no significant change in *tgfb1a* (Fig. 7D–G), consistent with a shift toward pro-inflammatory signaling and reduced expression of an M2-associated chemotaxis marker; however, transcript readouts alone do not establish macrophage state. No significant transcriptional changes were observed in the *tert* model, supporting distinct immunologic contexts across etiologies.

### 3.8 PMR Suppresses Proliferation Programs and Restores Hepatocyte Morphology In Vivo

**Figure 8:**
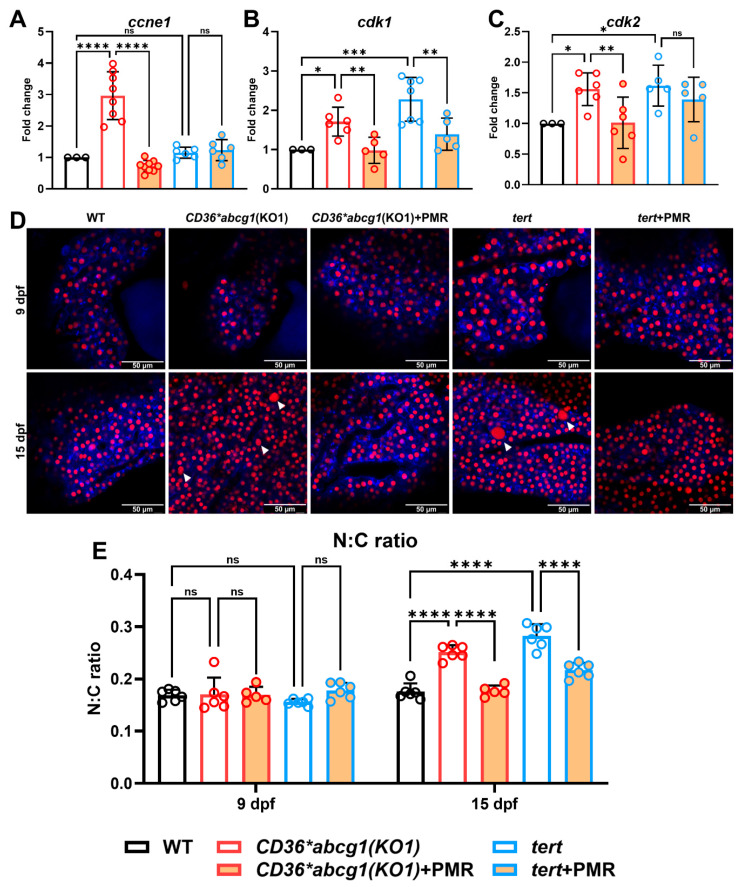
**PRS-OPT-nominated PMR dose suppresses proliferation markers and improves dysplasia-associated hepatocyte morphology in zebrafish HCC models.** (**A**–**C**) Hepatic qPCR of cell-cycle/proliferation markers *ccne1* (**A**), *cdk1* (**B**), and *cdk2* (**C**) in WT, the MASLD-HCC model Tg(*fabp10a:Palmitoyl-mTurquoise*, *H2A-mCherry*, *CD36; myl7:EGFP*); *abcg1(KO1)*, and the general HCC model Tg(*fabp10a:Palmitoyl-mTurquoise*, *H2A-mCherry*, *tert*; *myl7:EGFP*) treated with vehicle or the PRS-OPT–nominated PMR dose (propolis 1.4 × 10^−6^, metformin 13 μM, regorafenib 16.5 nM). (**D**) Representative confocal images of livers at 9 and 15 dpf. Palmitoyl-mTurquoise labels hepatocyte cytoplasm and H2A-mCherry labels nuclei; arrowheads indicate hepatocytes with enlarged nuclei consistent with dysplasia. Scale bars, 10 μm (shown on each micrograph). (**E**) Quantification of hepatocyte nuclear-to-cytoplasmic (N:C) ratio at 9 and 15 dpf. Individual larvae are overlaid with bars indicating mean ± SD; statistical comparisons are annotated on the plots (*p < 0.05; **p < 0.01; ***p < 0.001; and ****p < 0.0001; ns, not significant).

In MASLD-HCC larvae, a graded PMR dose series reduced the expression of cell-cycle regulators *ccne1*, *cdk1*, and *cdk2*, with the largest suppression observed at the highest dose tested (Fig. S9A–C). Notably, the PRS-OPT–nominated PMR regimen—selected based on immune endpoints—also downregulated these proliferation markers in the MASLD-HCC (Fig. 8A–C). In the *tert*-driven HCC model, which exhibits a highly proliferative phenotype, the same regimen significantly decreased *cdk1* but had little effect on *cdk2*, indicating context-dependent and partial responsiveness (Fig. 8A–C).

Finally, we quantified hepatocyte nuclear-to-cytoplasmic (N:C) ratio using membrane and nuclear fluorescent reporters. At 9 dpf, N:C ratios were comparable across groups. By 15 dpf, both MASLD-HCC and *tert* models showed elevated N:C ratios, consistent with dysplastic morphology. Treatment with the PRS-OPT–nominated PMR dose reduced N:C ratios toward WT levels in both models (Fig. 8D,E), supporting a restoration of hepatocyte cellular architecture in addition to the observed immune and transcriptional effects.

## 4 Discussion

### 4.1 PRS-OPT–Guided Optimization as a Precision Strategy for Efficacy and Safety

This study leverages PRS-OPT to nominate dosing windows for a three-component regimen—Taiwan propolis, metformin, and regorafenib—in HCC models under MASLD-like stress. Under FA exposure, PRS-OPT nominated a regimen predicted to achieve ~35% residual PLC/PRF/5 viability while maintaining ~81% THLE-2 viability; by contrast, the FA-free optimum that achieved near-complete PLC/PRF/5 suppression but reduced THLE-2 viability (~32%). Instead of relying on empirical grid searches or purely mechanistic frameworks that may miss non-linear interaction structure, PRS-OPT maps multidimensional dose–phenotype relationships and nominates hepatocyte-sparing, tumor-preferential regimens with a minimal experimental footprint. The PRS-OPT-optimized PMR reduced viability of two biologically distinct hepatoma lines (PLC/PRF/5 and HepG2) while sparing normal hepatocytes (THLE-2), and retained selectivity under FA exposure, a MASLD-like metabolic context. Together, these data illustrate how phenotype-driven modeling can delineate practical therapeutic windows that balance antitumor activity and hepatocyte tolerance in metabolically dynamic settings. 

Notably, the FA-dependent shift in the nominated optimum—most prominently the increase in metformin from 3 mM (FA-free) to 10 mM (FA-enriched)—is best interpreted as constraint-driven rather than as evidence that higher metformin is intrinsically superior under lipid stress. FA exposure altered both the fitted response surfaces and the feasible region imposed by the predefined hepatocyte-sparing constraint, such that dose sets that were near-optimal for tumor suppression in FA-free media no longer satisfied the THLE-2 viability requirement under FA. Within the experimentally bounded search space, PRS-OPT therefore moved toward a region in which metformin contributed to maintaining THLE-2 viability (or minimally impacting it) while preserving measurable tumor inhibition in lipid-loaded conditions. This shift is biologically plausible because FA loading can reprogram cellular energy/redox demands and drug responsiveness; however, the present study does not identify a single causal mechanism, and the metformin shift should be viewed as a model-guided consequence of altered phenotype-and-constraint geometry rather than a generalizable rule across MASLD contexts.

A key limitation is the absence of chromatographic fingerprinting of the propolis extract. Batch-related variability was mitigated by standardized extraction parameters (solvent, temperature/time, raw-to-solvent ratio) and use of a single batch across experiments; chemical fingerprinting and batch standardization remain required for translational development (see Section 4.7).

### 4.2 Mechanistic Context for Differential Death-Pathway Sensitivity

The divergent genetics of PLC/PRF/5 (HBV DNA integration, HBsAg secretion; TP53 R249S) and HepG2 (HBV-negative, TP53 wild-type, CTNNB1 exon-3 activation) provide a plausible basis for the distinct liabilities we observed (Section 3.4 and Section 3.5). PLC/PRF/5’s background is well documented (HBV integration/antigen production; TP53 R249S) [37,38], whereas HepG2 is widely recognized as TP53 wild-type with active β-catenin signaling and exon-3 CTNNB1 alteration driving stabilization [39], and is frequently used for metabolic/lipotoxic studies [40]. At a conceptual level, wild-type p53 can restrain cystine import via the SLC7A11 axis and modulate ferroptosis thresholds, while specific TP53 alterations dampen this checkpoint and bias cells toward classical apoptotic execution. Conversely, β-catenin signaling rewires hepatic metabolic/redox programs that intersect with ferroptosis determinants (e.g., lipid composition/ROS handling), consistent with the ferroptosis susceptibility often observed in HepG2 to system xC^−^/GPX4 pathway perturbation (e.g., erastin) [41,42]. These literature-supported axes align with our inhibitor and biomarker readouts—robust caspase activation and multi-pathway partial rescue in PLC/PRF/5 versus pronounced ferrostatin-1 rescue in HepG2—and provide a mechanistic context for the cell-type specificity of PMR cytotoxicity.

Pharmacologic rescue experiments showed that PMR does not converge on a single execution pathway. In PLC/PRF/5, blockade of apoptosis (Z-VAD-FMK), ferroptosis (ferrostatin-1), necroptosis (necrostatin-1), or autophagy (3-methyladenine) each partially restored viability, supporting engagement of multiple cell-death programs. In HepG2, only ferrostatin-1 produced meaningful rescue, supporting a ferroptosis-associated contribution (Fig. 4C). These observations are compatible with prior reports of ferroptosis–apoptosis crosstalk [43], in which lipid peroxidation can impair mitochondrial integrity and sensitize intrinsic apoptosis [44], and apoptotic regulators can influence lipid-ROS stress and ferroptosis susceptibility [45]. Consistent with such interplay, PLC/PRF/5 displayed time-dependent increases in cleaved caspase-3, cleaved PARP, and BAX, supporting caspase-dependent apoptosis that may be amplified by oxidative/lipid stress. By contrast, HepG2 showed limited induction of apoptotic markers together with a ferroptosis-1-responsive rescue profile, highlighting cell-type-specific liabilities that PMR may exploit. Direct biochemical measurements of lipid peroxidation and iron handling (e.g., BODIPY-C11 oxidation, MDA/4-HNE adducts, labile iron pool, and GPX4/SLC7A11 functional assays) were not performed; accordingly, ferroptosis is treated as a supported interpretation rather than a definitive assignment.

### 4.3 Apoptosis as a Dominant Pathway Is Shaped by Cell Type and Lipid Exposure

Flow cytometry demonstrated pronounced apoptotic induction in PLC/PRF/5 following PMR treatment, with marked increases in both early and late apoptotic populations. In contrast, HepG2 cells accumulated predominantly in early apoptosis, a pattern consistent with the immunoblot data (robust caspase-3 and PARP cleavage in PLC/PRF/5 versus modest BAX induction without clear downstream caspase/PARP activation in HepG2). FA supplementation, used to model MASLD lipotoxic stress, attenuated apoptotic signaling in both lines; PLC/PRF/5 retained a measurable apoptotic response. This divergence likely reflects cell line-specific differences in mitochondrial vulnerability, lipid metabolism, and redox homeostasis [46]. Consistent with this interpretation, saturated FAs have been reported to dampen apoptotic execution through adaptive stress response, including autophagy-mediated cytoprotection [47,48]. The present data indicate that PMR retains pro-death activity under FA exposure, particularly in PLC/PRF/5, although the relative contributions of apoptosis versus other programs likely vary by cell context. Collectively, these findings emphasize that tumor genotype and metabolic context jointly shape death-pathway engagement, supporting the need for context-aware dose and partner selection in MASLD-HCC.

### 4.4 Mechanistic Contribution of PMR Components

The observed biology is concordant with known activities of the PMR constituents. Metformin reprograms tumor metabolism via AMPK, interfaces with mTOR and stress pathways, has been linked to ferroptosis-associated signaling (e.g., via ATF4/STAT3), and reshapes the immune milieu (reducing Treg and MDSC function and augmenting CD8^+^ infiltration) [49,50]. Regorafenib, a multikinase inhibitor (VEGFR/PDGFR/FGFR/RAF), suppresses angiogenic and oncogenic signaling and prolongs survival in advanced HCC [26]. Taiwan propolis contributes complementary, multi-targeted activities; its flavonoid/phenolic repertoire (e.g., CAPE, chrysin, pinocembrin, galangin) can inhibit NF-κB, modulate p53 and BCL-2/BAX axes, and intersect with MAPK and PI3K/AKT to promote apoptosis and limit inflammatory survival signals [51,52,53,54]. Although individual constituents may have limited stability or bioavailability *in vivo*, the polyphenolic diversity may confer network-level robustness. Collectively, these mechanisms provide a plausible basis for PMR’s multimodal cytotoxicity, preserved selectivity for malignant cells, and context-sensitive performance under FA exposure.

### 4.5 Zebrafish Resolve In Vivo Immune Modulation and Enable Immune Endpoint Optimization

The MASLD-HCC zebrafish platform allowed real-time visualization of innate immune dynamics in a metabolically stressed liver. Consistent with human MASLD pathology, macrophages are central to inflammatory remodeling and tumor promotion [55,56,57,58]. This macrophage emphasis is also reflected in MASLD/MASH literature, where hepatic macrophages (resident Kupffer cells and recruited monocyte-derived macrophages) are frequently implicated as dominant drivers of lipotoxic inflammation and downstream tissue remodeling, whereas neutrophil involvement can be more context-, stage-, and trigger-dependent. In our model, PMR reduced hepatic macrophage density in a dose-dependent manner without a detectable change in neutrophil levels, supporting an innate response that is compartment-selective rather than globally immunosuppressive. qPCR suggested marker shifts—upregulation of *tnfa* and *il1b* with downregulation of *cxcr4b*—consistent with a transcript pattern compatible with increased pro-inflammatory signaling and reduced expression of an M2-associated chemotaxis marker [59]. Because transcript readouts alone do not establish macrophage state, these results are interpreted as supportive rather than definitive evidence of polarization. Together, the macrophage-specific modulation observed here aligns with a MASLD-like inflammatory context in which macrophages are prominent effectors, while the absence of a neutrophil signal should be interpreted as a negative finding under the present model conditions rather than evidence that neutrophils are irrelevant across MASLD-HCC settings. These immune-associated effects were prominent in MASLD-HCC and were not recapitulated in a *tert*-driven HCC model, underscoring etiology-dependent immune biology.

We extended PRS-OPT beyond cytotoxicity to immune endpoints, using hepatic macrophage density ratios as the optimization target. The model nominated PMR doses that minimized macrophage accumulation and these nominations were prospectively validated *in vivo*. This proof-of-concept supports phenotype-based multi-objective optimization, enabling regimen nomination that jointly considers tumor killing and TME-associated readouts—an important capability in MASLD-HCC, where metabolism, inflammation, and oncogenesis are tightly coupled.

### 4.6 Clinical Implications, Limitations, and Future Directions toward Precision Oncology in MASLD-HCC

The present work advances a systems framework that couples PRS-OPT with mechanistic assays and disease-relevant *in vivo* imaging to nominate combination regimens for MASLD-HCC. In cell-based and zebrafish MASLD-like settings, PRS-OPT-nominated PMR dosing windows showed tumor-preferential activity with hepatocyte sparing under lipid-rich conditions, were associated with reduced hepatic macrophage infiltration with a shift in polarization markers, suppressed proliferation programs, and improved dysplastic hepatocyte morphology. These convergent effects across tumor, immune, and metabolic readouts motivate further preclinical development in a disease context where steatosis, inflammation, and tumor evolution are tightly intertwined. Given physiological and pharmacokinetic differences between zebrafish and mammals, translational evaluation will require validation in mammalian MASLD-HCC models (e.g., diet-induced MASLD-HCC or MASLD-like xenograft backgrounds) with integrated efficacy and hepatotoxicity endpoints. PRS-OPT is also directly extensible to additional response layers, including patient-derived organoids, and tumor–immune co-cultures, and multi-omics readouts collected at nominated doses, to support biomarker-informed dose nomination and patient stratification. Consistent with this, the large international real-world REFINE phase 4 study (NCT03289273) confirmed the safety and effectiveness of regorafenib in a clinically diverse uHCC population, including non-viral etiologies such as MASLD-associated HCC, supporting the translational relevance of the regorafenib component in PMR [60].

### 4.7 Limitations of the Study

This study has several limitations. (i) Propolis characterization and exposure: Taiwan propolis extract (GoldWise Co., Ltd., Taiwan) was not chemically fingerprinted (e.g., by HPLC/UPLC/LC–MS), and natural batch-to-batch variability may influence bioactivity. Variability was mitigated by fixed extraction parameters (solvent, temperature/time, raw-to-solvent ratio) and use of a single, traceable batch across experiments. Chemical fingerprinting, batch standardization, and PK/PD characterization are required next steps for translational development. (ii) Model generalizability: Zebrafish enable rapid, high-content *in vivo* phenotyping but differ from mammals in hepatic physiology and drug ADME; validation in mammalian MASLD-HCC models remains warranted. (iii) Cell-death mechanism readouts: Inhibitor profiling and immunoblotting support ferroptosis–apoptosis crosstalk, yet lipid peroxidation and iron-handling biochemistry were not directly quantified (e.g., 4-HNE adducts, labile iron pool, GPX4 activity); these assays will be incorporated in follow-up studies. (iv) Scope of PRS-OPT: Although PRS-OPT reduced experimental burden and identified a hepatocyte-sparing, tumor-preferential PMR dose window, broader training sets—including patient-derived organoids, tumor–immune co-cultures, and multi-omics immune profiling—may further improve predictive fidelity and support biomarker-guided patient selection.

## 5 Conclusions

This study establishes a practical, multi-objective and context-aware strategy for regimen nomination in MASLD-associated HCC by coupling phenotypic response surface–guided optimization (PRS-OPT) with a complementary three-agent regimen (Taiwan propolis extract, metformin, and regorafenib). Rather than optimizing cytotoxicity alone, PRS-OPT explicitly integrates tumor suppression with a hepatocyte-sparing constraint under MASLD-like FA stress and can incorporate additional phenotypic objectives, including *in vivo* immune endpoints. Within the experimentally tested space, PRS-OPT nominated dosing windows that preserved hepatocyte viability under FA exposure while maintaining measurable antitumor activity, and these nominations were prospectively validated across cell models and in a zebrafish MASLD-HCC platform with macrophage-dominant immune modulation and improved hepatocyte morphology. The scientific value-added is the demonstration that interpretable response-surface modeling can prospectively “move” the optimal dose region when metabolic context shifts, thereby operationalizing precision dose nomination in a lipid-stressed setting that is often underrepresented in standard HCC optimization workflows.

These results motivate a focused translation path: (i) mechanistic deepening with direct lipid peroxidation and iron-handling assays alongside apoptosis/ferroptosis markers, (ii) orthogonal validation in mammalian MASLD-HCC models with integrated efficacy, hepatotoxicity, and PK/PD endpoints, and (iii) biomarker-guided early clinical exploration that jointly tracks metabolic state, vascular signaling, and immune signatures. More broadly, PRS-OPT provides a generalizable framework for biomarker-informed dose and combination nomination that can be extended to additional drug classes and patient-derived systems (organoids and tumor–immune co-cultures) to support stratified, evidence-guided refinement toward clinical translation.

## Data Availability

*Lead contact:* Further information and requests for resources and reagents should be directed to and will be fulfilled by the Lead Contact, Chiou-Hwa Yuh (see title page for email address). *Materials availability:* No new antibodies or mammalian cell lines were generated in this study. PLC/PRF/5 (BCRC Cat# 60223, RRID:CVCL_0485), HepG2 (BCRC Cat# 60025, RRID:CVCL_0027), and THLE-2 were obtained from the sources listed in the Key Resources Table and are available from the vendors. The transgenic zebrafish lines used in this study (e.g., *Tg(mpeg:mCherry; mpx:EGFP)*, *Tg(fabp10a:Palmitoyl-mTurquoise, H2A-mCherry)*, and derivative crosses involving *fabp10a:CD36; abcg1(KO1) or fabp10a:tert*) can be shared for non-commercial academic use upon completion of a material transfer agreement (MTA) and in compliance with local regulations on animal transfer. The Taiwan propolis ethanol extract used in this work was prepared from a single authenticated batch under standardized conditions; limited research quantities can be provided upon reasonable request and completion of an MTA. (See “Limitations” in the Discussion regarding the lack of chromatographic fingerprinting of this batch). *Data and code availability:* All data supporting the findings are available within the main text and Supplementary Information. Raw data (e.g., uncropped immunoblots, original confocal image stacks, Oil Red O quantifications) and zebrafish imaging/analysis spreadsheets will be deposited in an open repository (e.g., Mendeley Data) upon acceptance; until then, they are available from the Lead Contact upon reasonable request. Analyses were performed using a custom-built Artificial Intelligence-Phenotypic Response Surface optimization platform (PRS-OPT) implemented in a Google Colaboratory environment running Python 3. Data processing and numerical computations were performed using pandas and numpy. Ordinary Least Squares (OLS) regression models were implemented using statsmodels.formula.api.ols from the statsmodels package. Visualization of the multi-dimensional response surfaces and validation plots was accomplished using matplotlib and plotly. The final dosage optimization was conducted using the ‘Sequential Least Squares Programming’ (SLSQP) algorithm from the scipy.optimize library. The analysis code and additional details required to reanalyze the data are available from the Lead Contact upon reasonable request.

## Supplementary Materials

The supplementary material is available online at https://www.techscience.com/doi/10.32604/or.2026.074145/s1.

## Abbreviations and Nomenclature

3-MA	3-methyladenine
ADME	absorption, distribution, metabolism, and excretion
AMPK	AMP-activated protein kinase
ATF4	activating transcription factor 4
BAX	BCL2-associated X protein
BODIPY 581/591 C11 (BODIPY-C11)	lipid peroxidation probe
BSA	bovine serum albumin
CCK-8	Cell Counting Kit-8
CD8	cluster of differentiation 8
DMEM	Dulbecco’s modified Eagle medium
DMSO	dimethyl sulfoxide
dpf	days post-fertilization
E3	E3 embryo medium
EGFP	enhanced green fluorescent protein
FA	fatty acid(s)
FBS	fetal bovine serum
Fer-1	ferrostatin-1
FGFR	fibroblast growth factor receptor
FITC	fluorescein isothiocyanate
HCC	hepatocellular carcinoma
Hep3B	human hepatoma cell line
HepG2	human hepatoma cell line
hpf	hours post-fertilization
HPLC	high-performance liquid chromatography
ICI	immune checkpoint inhibitor(s)
IFN-γ	interferon gamma
IL-4	interleukin 4
IL-8	interleukin 8
JNK	c-Jun N-terminal kinase
KO	knockout
LC–MS	liquid chromatography–mass spectrometry
LPS	lipopolysaccharide
MASH	metabolic dysfunction–associated steatohepatitis
MASLD	metabolic dysfunction–associated steatotic liver disease
MDA	malondialdehyde
MDSC	myeloid-derived suppressor cell(s)
N:C	nuclear-to-cytoplasmic ratio
NAC	N-acetylcysteine
Nec-1	necrostatin-1
OA	oleic acid
OLS	ordinary least squares
ORO	Oil Red O
PA	palmitic acid
PARP	poly(ADP-ribose) polymerase
PBS	phosphate-buffered saline
PDGFR	platelet-derived growth factor receptor
PI	propidium iodide
PK/PD	pharmacokinetics/pharmacodynamics
PLC/PRF/5	human hepatoma cell line
PMA	phorbol 12-myristate 13-acetate
PMR	propolis–metformin–regorafenib regimen
PRS	phenotypic response surface
PRS-OPT	phenotypic response surface–guided optimization
RAF	RAF kinase family
ROS	reactive oxygen species
RT	room temperature
SD	standard deviation
SLC7A11	cystine/glutamate transporter subunit xCT
SLSQP	sequential least-squares quadratic programming
STAT3	signal transducer and activator of transcription 3
Tg	transgenic
THLE-2	immortalized human hepatocyte line
TME	tumor microenvironment
Treg	regulatory T cell(s)
UPLC	ultra-performance liquid chromatography
VEGFR	vascular endothelial growth factor receptor
VIF	variance inflation factor
WT	wild type
Z-VAD-FMK	pan-caspase inhibitor
