# Supplementary material for: Phenotypic Response Surfaces–Guided Optimization (PRS-OPT) of Propolis-Metformin-Regorafenib Combination Therapy for MASLD-Associated Hepatocellular Carcinoma
Source: Oncol Res. 2026 May 21;34(6):25. doi: 10.32604/or.2026.074145 (PMC13227599; doi:10.32604/or.2026.074145)
Supplement: Supplementary file 1 [file OncolRes-34-74145-s001.zip › Supplementary_Files.docx]

**Supplementary Information**

**Supplementary Methods S1. Cell culture, authentication, and routine quality control (expanded)**

**S1.1 Cell lines and provenance**

PLC/PRF/5 (BCRC, 60223; RRID:CVCL_0485) and HepG2 (BCRC, 60025; RRID:CVCL_0027) were obtained from the Bioresource Collection and Research Center (BCRC, Hsinchu, Taiwan). THLE-2 (ATCC, CRL-2706; RRID:CVCL_3803) and THP-1 (ATCC, TIB-202; RRID:CVCL_0006) were obtained from ATCC (Manassas, VA, USA).

**S1.2 Authentication and contamination testing**

Cell identity was confirmed by short tandem repeat (STR) profiling (when applicable) and cultures were routinely confirmed mycoplasma-free using the LookOut® One-Step Mycoplasma PCR Detection kit (Applied Biological Materials Inc. [abm], G238, Richmond, BC, Canada) according to the manufacturer’s instructions. Cells were used within a controlled passage window to minimize drift (record passage ranges in lab notebook and/or state typical range in manuscript).

**S1.3 Routine culture conditions and passaging**

PLC/PRF/5 and HepG2 were maintained in Dulbecco’s modified Eagle medium (DMEM, Gibco, Thermo Fisher Scientific, 12100046, Waltham, MA, USA) supplemented with 10% FBS (Gibco, 16000-044) and 1% penicillin–streptomycin (P/S; Gibco, 15140-122). THLE-2 were maintained in BEGM® BulletKit (Lonza CC-3170, Walkersville, MD, USA) prepared from BEBM® basal medium (Lonza, CC-3171) and SingleQuots® supplements (Lonza, CC-4175), prepared without gentamicin/amphotericin B and epinephrine and supplemented with epidermal growth factor (EGF, Merck Millipore, 01-107/01-407, Burlington, Massachusetts, USA, final 5 ng/mL), phosphoethanolamine (Sigma-Aldrich, P0503, St. Louis, MO, USA, final 70 ng/mL), 10% fetal bovine serum (FBS, , Gibco, 11965-092) and 1% penicillin–streptomycin (P/S, Gibco, 15140-122).

Cells were maintained at 37 °C in a humidified incubator with 5% CO₂. For passaging, cultures were washed with PBS, detached using TrypLE™ Express (Gibco Cat. #12604021), neutralized with complete DMEM (Gibco, 12100046) or BEBM (Lonza CC-3170) medium, centrifuged, and reseeded at a 1:3 split ratio for routine subculture. For viability assays, cells were plated at 3,000 cells/well in 96-well plates (as specified in Section 2.4 and figure legends). Cells were routinely examined by phase-contrast microscopy for morphology and confluence before experimental setup.

**S1.4 THP-1 differentiation and polarization (expanded)**

THP-1 were maintained in RPMI-1640 (Gibco Cat. #31800022) with 10% FBS and 1% PS. Differentiation into macrophage-like cells was induced with 100 nM PMA (Sigma-Aldrich Cat. No. 79346; CAS: 16561-29-8) for 24 h. Cells were then washed and rested in PMA-free medium as needed (optional rest period may be specified if used). Polarization was performed for 48 h as follows:

- M1: IFN-γ 2 ng/mL (R&D Systems, 285-IF, Minneapolis, MN, USA, final 2 ng/mL) + LPS 100 ng/mL (Sigma-Aldrich, L2630, final 100 ng/mL)
- M2: IL-4 20 ng/mL (Sigma-Aldrich, I4269)

**Supplementary Methods S2. Taiwan propolis: source, extraction, stock preparation, storage, and working solutions (expanded)**

**S2.1 Source and traceability**

Raw Taiwan propolis (Apis mellifera; Taiwan origin) was obtained as a research-grade material from GoldWise Co., Ltd. (Taipei City, Taiwan). A single preparation batch was used for all experiments. Batch ID, receipt date, and extraction yield were documented internally for traceability.

**S2.2 Extraction procedure**

Propolis was extracted in 75% (v/v) ethanol at a raw-to-solvent ratio of 1 kg:5 L (equivalent to 200 mg raw material/mL solvent). The mixture was agitated at 40 °C (80 rpm) for 30–60 min. The extract was clarified by coarse filtration and subsequently sterile-filtered through a 0.22 µm filter. The resulting extract stock was defined as 200 mg/mL (w/v; raw-material equivalents).

**S2.3 Storage and handling**

Stock was stored protected from light at room temperature. Immediately before use, working solutions were prepared by diluting the stock into culture medium to the indicated concentrations. Final ethanol content was matched across all treatment groups and maintained at ≤0.1% (v/v), including vehicle controls.

**S2.4 Unit conversion for reporting**

Propolis dosing was reported as a fold-dilution of the 200 mg/mL stock and optionally converted to raw-equivalent mass concentration. For example, 1×10⁻⁴ corresponds to 20 µg/mL and 0.3×10⁻⁴ corresponds to 6 µg/mL (raw-equivalent).

**Supplementary Methods S3. Fatty acid (FA) preparation and FA-enriched culture conditions (expanded)**

**S3.1 Reagents**

Oleic acid (OA; Sigma-Aldrich/Merck Cat. #O1008) and palmitic acid (PA; Sigma-Aldrich/Merck Cat. #P0500) were prepared as BSA-conjugated stocks using fatty acid–free BSA (Sigma-Aldrich/Merck Cat. #A8806). Ethanol served as the initial solvent for OA/PA dissolution.

**S3.2 Preparation of BSA-conjugated FA stocks**

OA and PA were dissolved in ethanol and conjugated to 5% (w/v) fatty acid–free BSA prepared in serum-free DMEM (for PLC/PRF/5 and HepG2) or BEBM (for THLE-2), yielding 6 mM OA and 2.5 mM PA stock solutions. Aliquots were stored at −20 °C and thawed immediately before use. Stocks were mixed thoroughly to ensure homogeneity prior to dilution.

**S3.3 Working FA medium and vehicle controls**

FA-enriched working medium was freshly prepared by diluting stocks into the corresponding culture medium to final concentrations of 0.5 mM OA + 0.25 mM PA. FA exposure was applied concurrently with drug treatment unless otherwise specified. Vehicle-matched controls contained fatty acid–free BSA and ethanol at concentrations matched to FA conditions, with ethanol maintained at ≤0.1% (v/v) across all groups.

**Supplementary Methods S4. Cell viability assay (CCK-8): plating, treatment schedule, and calculation (expanded)**

Cells were seeded in 96-well plates at 3,000 cells/well and allowed to adhere overnight. Treatments (single agents or PMR combinations) were applied ± FA for the indicated duration (typically 72 h unless stated otherwise in figure legends). CCK-8 reagent (Dojindo Cat. #CK04) was added at 10 µL per well and incubated for 2 h at 37 °C, then absorbance at 450 nm was measured. Background subtraction was performed using medium-only wells processed identically. Viability was reported as percent of vehicle control within the same FA condition.

**Supplementary Methods S5. PRS-OPT dose optimization (expanded)**

Mean viability values from *in vitro* combination experiments were used as inputs to the PRS-OPT workflow to fit multidrug dose–response surfaces and nominate *Propolis–Metformin–Regorafenib* (PMR) dose sets that prioritize tumor suppression while sparing normal hepatocytes. For *in vivo* optimization, zebrafish imaging-derived hepatic macrophage infiltration metrics (macrophage density ratios at 15 days post-fertilization [dpf] relative to 9 dpf or 12 dpf) were used as model outputs to nominate PMR doses that minimize macrophage accumulation.

**Rationale for using hepatic macrophage density ratios**. In larval zebrafish, macrophages are the primary innate immune responders and rapidly accumulate in tissues undergoing inflammatory stress or injury. Accordingly, hepatic macrophage infiltration has been widely used as a quantitative surrogate readout of liver immune activation in zebrafish models of metabolic liver stress and hepatotoxicity. In this study, we operationalized inflammatory activation/inflammatory toxicity as the change in liver-associated macrophage density over time, quantified as a within-fish ratio (15 dpf divided by the corresponding baseline at 9 or 12 dpf). This longitudinal ratio reduces confounding from inter-individual differences in liver size, baseline macrophage abundance, and imaging intensity, enabling more robust comparison of immune activation across diet and drug-treatment groups within the experimentally tested PMR dose space.

**Workflow overview.** PRS-OPT proceeded in five steps: (i) define inputs/outputs (drug concentrations as inputs; viability or macrophage-density ratios as outputs), (ii) fit an interpretable quadratic response-surface model to capture main effects and drug–drug interactions, (iii) evaluate model adequacy using prespecified diagnostics and robustness checks, (iv) perform constrained optimization within the experimentally tested bounds to nominate dose sets that satisfy explicit biological constraints, and (v) prospectively validate nominated dose sets in independent *in vitro* experiments and zebrafish assays. A detailed mathematical formulation, solver settings, and implementation notes are provided in Supplementary Methods.

To capture nonlinear dose effects and drug-drug interactions, concentrations were mean-centered and expanded to a second-order polynomial basis comprising linear terms, squared terms, and pairwise interaction terms. Quadratic response-surface models were then fit by ordinary least squares (OLS) separately for PLC/PRF/5 and THLE-2, yielding one response surface per cell type. Model adequacy was evaluated using predicted-versus-observed plots and the coefficient of determination (*R*^2^), and predictive robustness/uncertainty was further quantified by K-fold cross-validation (cross-validated RMSE) and nonparametric bootstrap (confidence intervals for coefficients and/or predictions), with full details and results provided in the Supplementary Information.

**Assessment of OLS assumptions and rationale for OLS.** Standard OLS assumptions were evaluated using residual-based diagnostics for normality (Shapiro–Wilk test and Q–Q plots), homoscedasticity (Breusch–Pagan test and residuals-versus-fitted plots), and error independence (Durbin–Watson statistic and residuals-versus-run-order plots); multicollinearity among polynomial terms was assessed using variance inflation factors (VIFs) on the mean-centered design matrix. Diagnostic plots and summary statistics are provided in the Supplementary Information (SI1–SI3) with full implementation details in Supplementary Methods S6. OLS was retained primarily for interpretability and transparent constrained dose nomination within the experimentally tested bounds, and model conclusions were additionally supported by cross-validation and bootstrap-based uncertainty estimation.”

Dose nomination was performed by constrained multi-objective optimization using Sequential Least Squares Programming (SLSQP) within experimentally defined bounds: the primary objective minimized predicted PLC/PRF/5 viability, and hepatocyte sparing was operationalized by selecting solutions that maximize predicted THLE-2 viability among near-optimal tumor-killing solutions (within a small tolerance of the minimum predicted PLC/PRF/5 viability). Near-optimal tumor-killing solutions were defined using an ε-optimal criterion: predicted PLC/PRF/5 viability ≤ 1.05 × V_min, where V_min is the minimum predicted PLC/PRF/5 viability within the experimentally tested bounds. Final dose nomination selected the solution that maximized predicted THLE-2 viability among this ε-optimal subset. For *in vivo* immune optimization, the objective minimized the predicted macrophage-density ratio within the experimentally tested PMR bounds using the same response-surface/constraint framework. All nominated dose sets represent model-dependent optima within the tested dose space and are not intended to imply clinical optima.

**Supplementary Methods S6. OLS assumption checks and uncertainty diagnostics for PRS-OPT response-surface models**

**S6.1 Model fitting and residual definition.**
For each condition (PLC5 non-FA, THLE-2 non-FA, PLC5 FA, THLE-2 FA), viability data were fit using a second-order (quadratic) response-surface model by ordinary least squares (OLS). Residuals were computed as *e* = *y* − ŷ, where *y* is observed viability and ŷ is model-predicted viability.

**S6.2 Polynomial feature construction and structural collinearity mitigation.**
To capture nonlinear dose effects and pairwise drug–drug interactions, predictors were expanded to include linear terms, squared terms, and pairwise interaction terms. To reduce structural multicollinearity introduced by polynomial expansion, drug concentrations were mean-centered prior to generating squared and interaction terms.

**S6.3 Predictor scaling sensitivity analysis.**
Because drug concentrations span different numerical ranges, models were fit using concentrations in their original units within the experimentally defined bounds. As a sensitivity analysis, predictors were also z-score standardized prior to refitting; standardization did not materially change model adequacy metrics or the location of model-based optima (reported in Supplementary Information).

**S6.4 Multicollinearity assessment (Variance Inflation Factors).**
Multicollinearity among main effects and interaction terms was evaluated using variance inflation factors (VIFs) calculated on the model design matrix. Across the four fitted models, no evidence of severe multicollinearity was observed (maximum VIF < 3 for all models; Supplementary diagnostic summary).

**S6.5 Residual normality (Shapiro–Wilk) and visual diagnostics.**
Residual normality was assessed using the Shapiro–Wilk test (function scipy.stats.shapiro from the SciPy library), with *p* < 0.05 interpreted as evidence against normality. In addition, Q–Q plots and residual histograms were generated (Supplementary Information Figure S1) to visually evaluate departures from normality.

**S6.6 Homoscedasticity (Breusch–Pagan) and residual–fitted plots.**
Homoscedasticity (constant residual variance) was tested using the Breusch–Pagan procedure (implemented in Statsmodels as statsmodels.stats.diagnostic.het_breuschpagan), with *p* < 0.05 interpreted as evidence of heteroscedasticity. Residuals versus fitted-value plots were also examined (Supplementary Information Figure S2) to identify variance trends or systematic structure.

**S6.7 Independence of errors (Durbin–Watson) and residual–order plots.**
Error independence was assessed using the Durbin–Watson statistic (statsmodels.stats.stattools.durbin_watson). Residuals were additionally plotted against experiment/run order (Supplementary Information Figure S3) to screen for autocorrelation or batch/sequence effects.

**S6.8 Predictive robustness via K-fold cross-validation (CV).**
Predictive stability was quantified using K-fold cross-validation implemented with the scikit-learn library (sklearn.model_selection.KFold). For each fold, models were trained on K−1 partitions and evaluated on the held-out partition; performance was summarized as cross-validated root mean squared error (RMSE), averaged across folds. Observed RMSE values across the four models are reported in the Supplementary diagnostic summary.

**S6.9 Parameter uncertainty via bootstrap resampling.**
To characterize coefficient uncertainty, nonparametric bootstrap resampling was performed using scikit-learn (sklearn.utils.resample). Models were refit on resampled datasets to generate empirical coefficient distributions and 95% confidence intervals (CIs). Effects with CIs crossing zero were interpreted as weak or uncertain contributions, whereas CIs not crossing zero indicated more stable directional effects. Bootstrap summaries are provided in the Supplementary Information.

**Supplementary Methods S7. Oil Red O staining and quantification (expanded)**

**S7.1 Reagent preparation**

Oil Red O (ORO) stock was prepared by dissolving 35 mg ORO dye (Sigma-Aldrich Cat. #O-0605) in 10 mL 100% isopropanol, followed by filtration (0.22 µm). Stock was stored at room temperature. Working ORO solution was prepared fresh by mixing stock with ddH₂O at 3:2 (v/v), incubating 20 min at room temperature, and filtering before use.

**S7.2 Staining protocol**

Cells were seeded in 24-well plates at 15,000 cells/well, treated ± FA ± drugs for 72 h, washed twice with PBS, and fixed in 10% formalin (≥1 h, room temperature). Cells were washed with ddH₂O, equilibrated in 60% isopropanol for 5 min, air-dried, and stained with ORO working solution (500 µL/well) for 10 min. Cells were washed 4× with ddH₂O and imaged.

**S7.3 Elution-based quantification**

For quantification, ORO was eluted with 100% isopropanol (500 µL/well, 10 min, gentle shaking). Eluates were transferred to 96-well plates and absorbance at 500 nm was measured, using 100% isopropanol as blank.

**Supplementary Methods S8. Inhibitor pretreatment (expanded)**

Cells were plated at 3,000 cells/well (96-well plates). Pathway inhibitors were applied for 4 h, followed by co-treatment with PMR plus the same inhibitor for 24 h prior to CCK-8 readout. Inhibitors included: NAC (1 mM; TargetMol Cat. #T5518), Z-VAD-FMK (50 µM; TargetMol Cat. #T6013), ferrostatin-1 (10 µM; TargetMol Cat. #T6500), necrostatin-1 (50 µM; TargetMol Cat. #T1847), and 3-methyladenine (10 µM; TargetMol Cat. #T1879). Stocks were prepared in DMSO (NAC in water), stored at −20 °C, and diluted into medium immediately before use. Final DMSO was vehicle-matched across groups.

**Supplementary Methods S9. Annexin V/PI apoptosis assay and flow cytometry gating (expanded)**

Cells were seeded in 6-well plates (2.5 × 10⁵ cells/well) and treated with PMR ± FA for 48 h. Cells were washed with PBS, detached with TrypLE™ Express, resuspended in 1× Binding Buffer, stained with Annexin V–FITC (5 µL) and PI (5 µL) (Abcam Cat. #ab14085) for 5 min in the dark at room temperature, and analyzed immediately by flow cytometry.

Quadrants were defined using unstained, single-stained, and vehicle controls as appropriate. Populations were quantified as: viable (Annexin V⁻/PI⁻), early apoptotic (Annexin V⁺/PI⁻), late apoptotic (Annexin V⁺/PI⁺), and PI-only (Annexin V⁻/PI⁺).

**Supplementary Methods S10. Western blotting: lysis, electrophoresis, transfer, detection, and densitometry (expanded)**

**S10.1 Lysis and protein preparation**

Cells were treated with PMR ± FA for 0, 24, or 48 h. Cells were washed with cold PBS and lysed in RIPA buffer composed of 150 mM NaCl, 5 mM EDTA, 25 mM Tris-HCl (pH 7.6), 1% NP-40, 1% sodium deoxycholate, and 0.1% SDS, supplemented with protease inhibitor cocktail (MedChemExpress Cat. #HY-K0010) and phosphatase inhibitors (MedChemExpress Cat. #HY-K0021, #HY-K0022, #HY-K0023). Lysates were clarified by centrifugation and supernatants were collected.

Protein concentration was determined using BCA assay (Thermo Fisher, 23227). Samples were normalized to equal protein amounts (typically 30 µg per lane), mixed with appropriate loading buffer and reducing agent, heated as needed, and loaded for electrophoresis.

**S10.2 Gel electrophoresis and transfer**

Proteins were resolved on Bis-Tris gels (MOEKO BIO, P20412-010, Taipei, Taiwan) and transferred to PVDF membranes (0.22 µm).

**S10.3 Blocking, antibodies, and detection**

Membranes were blocked in 3% BSA/TBST (TBST: Protech Technology Enterprise, BF204, Taipei, Taiwan) for 1 h at room temperature, incubated with primary antibodies at 4 °C overnight, washed with TBST (3×5 min), incubated with HRP-conjugated secondary antibodies for 1 h at room temperature, washed, and developed using chemiluminescent substrate (T-Pro LumiLong Plus, JT96-K004M, New Taipei City, Taiwan). Signals were captured using a ChemiDoc™ MP (Bio-Rad, Hercules, CA, USA).

**S10.4 Densitometry**

Band intensities were quantified using ImageJ (v2.16.0; National Institutes of Health [NIH], Bethesda, MD, USA). Targets were normalized to β-actin and expressed relative to the time 0 control within each cell line/condition.

**Supplementary Methods S11. Zebrafish husbandry, lines, imaging, and quantification (expanded)**

**S11.1 Husbandry and ethics**

Zebrafish were maintained in the Taiwan Zebrafish Core Facility at NHRI (AAALAC-accredited) at 28 °C with a 14:10 h light:dark cycle. All procedures were approved by NHRI-IACUC (NHRI-IACUC-113155-A). Embryos/larvae were raised in E3 medium and staged by dpf unless otherwise indicated.

**S11.2 Lines (expanded)**

Transgenic lines Tg(mpeg:mCherry; mpx:EGFP) and Tg(fabp10a:Palmitoyl-mTurquoise, H2A-mCherry) and were crossed with laboratory MASLD-HCC and general HCC lines to generate Tg(mpeg:mCherry; mpx:EGFP; fabp10a:CD36; myl7:EGFP); abcg1(KO1); Tg(mpeg:mCherry; mpx:EGFP; fabp10a:tert; myl7:EGFP); Tg(fabp10a:Palmitoyl-mTurquoise, H2A-mCherry, CD36; myl7:EGFP); abcg1(KO1) and Tg(fabp10a:Palmitoyl-mTurquoise, H2A-mCherry, tert; myl7:EGFP). Reporters enabled visualization of: macrophages (mpeg:mCherry), neutrophils (mpx:EGFP), hepatocyte membranes (fabp10a:Palmitoyl-mTurquoise), nuclei (fabp10a:H2A-mCherry), and myocardium (myl7:EGFP) as an anatomical reference.

**Supplementary Methods S12. Embryotoxicity (expanded)**

Embryos were collected at ~2 h post-spawning, screened to remove non-viable embryos, and maintained in E3 buffer (5 mM NaCl, 0.17 mM KCl, 0.33 mM CaCl₂, 0.33 mM MgSO₄; pH 7.2) at 28 °C. Taiwan propolis was applied from 3 h post-fertilization (hpf) to 5 dpf with daily renewal. Survival and gross morphological abnormalities were recorded daily by stereomicroscopy. Sample sizes per concentration were **n = 10 embryos**, repeated across **3 independent clutches/experiments**.

**Supplementary Methods S13. Diet, drug exposure, randomization, and blinding (expanded)**

To inhibit pigmentation for imaging, larvae were maintained in PTU-supplemented E3 (0.002% PTU from 2 dpf; 0.003% from 9 dpf). Until 5 dpf, larvae were housed in Petri dishes; thereafter, cohorts were transferred to tanks (n ≈ 40 per tank) and fed a standardized schedule (paramecia and *Artemia*). *CD36;abcg1(KO1)* lines received a high-fat diet (24% fat) , whereas other lines received a standard diet (LD100, 12% fat). Drug doses were selected based on embryotoxicity testing. Stock solutions were prepared in ddH₂O or DMSO and diluted in E3 for daily bath treatment in 24-well plates (one larva per well) from 10 dpf to 15 dpf with daily renewal. Larvae were randomly allocated to treatment groups. Confocal images were acquired under standardized settings (Section 2.14). Images were coded prior to analysis, and ImageJ-based quantification was performed with the analyst blinded to treatment group.

**Supplementary Methods S14. Confocal microscopy (expanded)**

Larvae were anesthetized with 1× tricaine (MS-222, Sigma-Aldrich, Cat. # 108321-42-2) and mounted in 1.25% low-melting agarose (Zymeset, Taipei, Taiwan, Cat. #BAG102) in PTU/E3 on glass-bottom dishes. *Tg(mpeg:mCherry; mpx:EGFP)*, derivatives were imaged at 9, 12, and 15 dpf on a Leica TCS SP5 confocal microscope (Leica Microsystems, Wetzlar, Germany). Tg(fabp10a:Palmitoyl-mTurquoise, H2A-mCherry) derivatives were imaged at 9 and 15 dpf on a Leica Stellaris 8 using identical acquisition settings within each experiment. Images were analyzed in ImageJ. Hepatic macrophage and neutrophil infiltration were quantified within a predefined liver ROI, and densities were normalized to liver area to account for inter-individual variation in liver size (reported as cells or fluorescent area per liver area; see Supplementary Methods). The macrophage infiltration ratio was defined as the area-normalized hepatic macrophage density at 15 dpf divided by that at 9 dpf (or 12 dpf, as indicated). The same approach was applied to neutrophil density ratios.

**Supplementary Methods S15. RNA extraction, cDNA synthesis, and qPCR (expanded)**

**S15.1 RNA extraction from zebrafish larvae**

Total RNA was isolated from individual larvae using NucleoSpin® RNA (MACHEREY-NAGEL GmbH & Co. KG, 740955.50, Düren, Germany) with minor modifications. Individual larvae were homogenized in 350 µL RA1 buffer supplemented with 3.5 µL β-mercaptoethanol using 0.5-mm RNase-free zirconium beads in a Bullet Blender. Lysates were clarified using NucleoSpin® Filters (11,000 × *g*, 1 min, 4 °C), mixed with 70% ethanol (1:1, v/v), loaded onto columns, and subjected to on-column DNase digestion (95 µL, 30–60 min). Columns were washed per kit instructions and RNA was eluted in 20 µL RNase-free water (13,000 × *g*, 2 min). RNA was quantified by NanoDrop and stored at −80 °C.

**S15.2 cDNA synthesis**

cDNA was synthesized from 1 µg RNA using iScript™ cDNA Synthesis Kit (Bio-Rad Laboratories, 1708890, Hercules, CA, USA) in 20 µL reactions under the cycling conditions: 25 °C 5 min; 46 °C 20 min; 95 °C 1 min; hold at 4 °C. cDNA was stored at −20 °C.

**S15.3 qPCR**

qPCR was performed on QuantStudio™ 5 using SYBR Green (Thermo Fisher Scientific, 4385618, Waltham, MA, USA) in 384-well plates (10 µL/well: 5 µL 2× SYBR, 1.2 µL primers at 2.5 µM each, 3.8 µL diluted cDNA containing 40 ng). Cycling: 95 °C 3 min; 40 cycles of 95 °C 1 s and 60 °C 20 s; melt curve: 95 °C 15 s, 50 °C 1 min, 95 °C 15 s. Relative expression was calculated using ΔΔCt method with actin as the reference; based on assay calibration, the amplification efficiency was 1.94 per cycle (≈94%), and fold change was computed as 1.94^−ΔΔCt^. Primer sequences are listed in Table S1.

**Table S1. The primer information for qPCR analysis**

| Primer name | Accession | Sequencing |
| --- | --- | --- |
| *actin*-F | NM_131031.1 | 5’-CTCCATCATGAAGTGCGACGT-3’ |
| *actin*-R |  | 5’-CAGACGGAGTATTTGCGCTCA-3’ |
| *ccne1*-F | NC_007118.7 | 5’-CATGCCAAGCAAGAAAGTGCTA-3’ |
| *ccne1*-R |  | 5’-GTGCTGGGAACACCTTCAGT-3’ |
| *cdk1*-F | NM_212564.2 | 5’-CTCTGGGGACCCCTAACAAT-3’ |
| *cdk1*-R |  | 5’-CGGATGTGTCATTGCTTGTC-3’ |
| *cxcr4b*-F | NM_131834.1 | 5’-CAATGGACTTGTGGTGCTTG-3’ |
| *cxcr4b*-R |  | 5’-CACAGAAATCCCCCAAAATG-3’ |
| *il1b*-F | NM_212844.2 | 5’-TGGACTTCGCAGCACAAAATG-3’ |
| *il1b*-R |  | 5’-GTTCACTTCACGCTCTTGGATG-3’ |
| *tgfb1a*-F | NM_182873.1 | 5’-CAACCGCTGGCTCTCATTTGA-3’ |
| *tgfb1a*-R |  | 5’-ACAGTCGCAGTATAACCTCAGCT-3’ |
| *tnfa*-F | NM_212859.2 | 5’-TACGGAGGCAAAAAGCCACT-3’ |
| *tnfa*-R |  | 5’-AGAAGTGCTGTGGTCGTGTC-3’ |


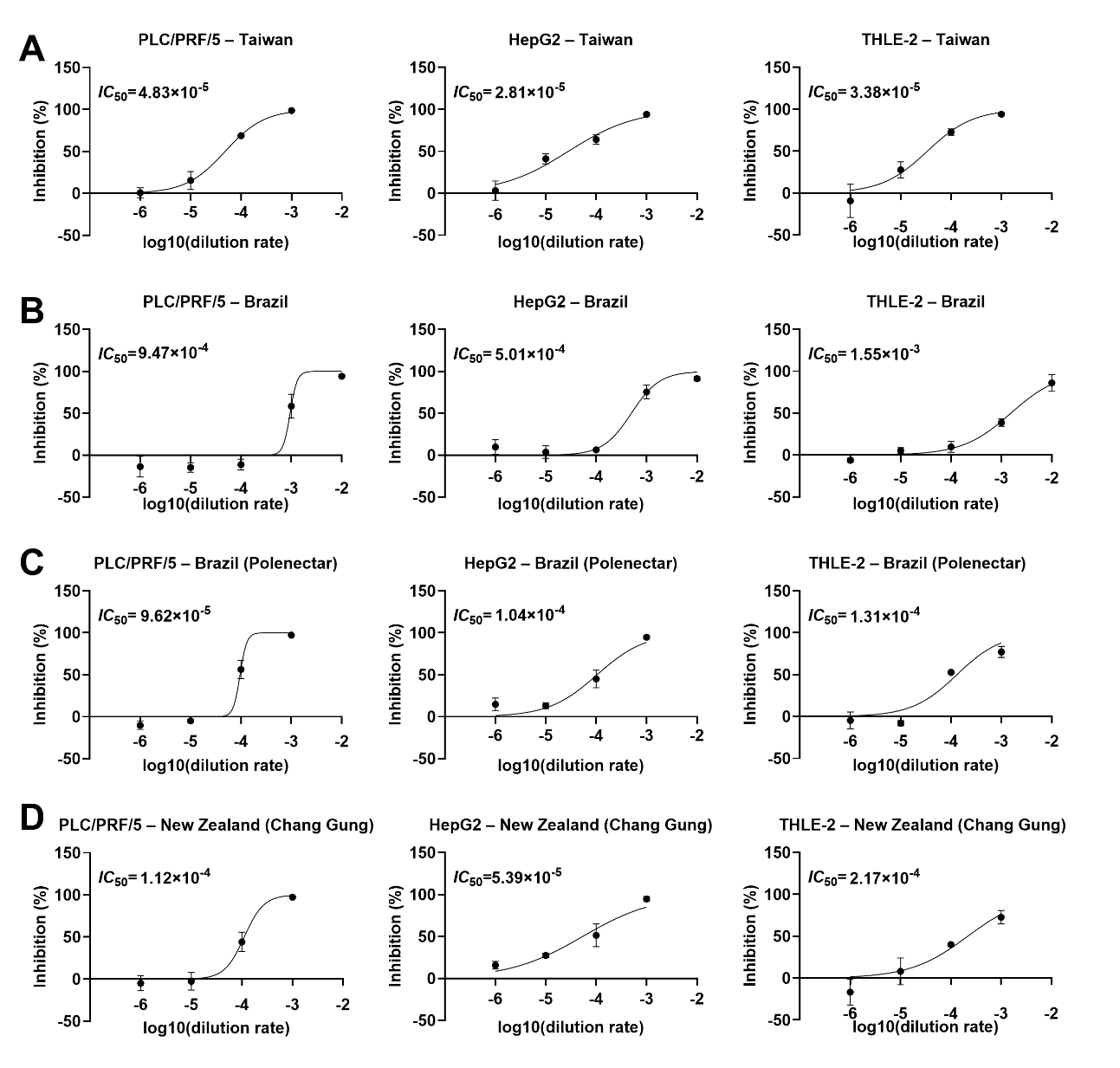


**Figure S1. Source-dependent antiproliferative activity of propolis extracts across liver cell models.** PLC/PRF/5, HepG2, and THLE-2 cells were treated for 72 h with propolis extracts from four sources, and dose–response curves were fitted to estimate IC₅₀ values. Panels show extracts from (**A**) Taiwan, (**B**) Brazil, (**C**) Polenectar (Brazil; commercial product), and (**D**) New Zealand (Chang Gung Biotech; commercial product). The x-axis indicates log10(dilution factor) relative to each extract stock, and the y-axis shows growth inhibition (%). Points represent mean ± SD from three independent experiments.


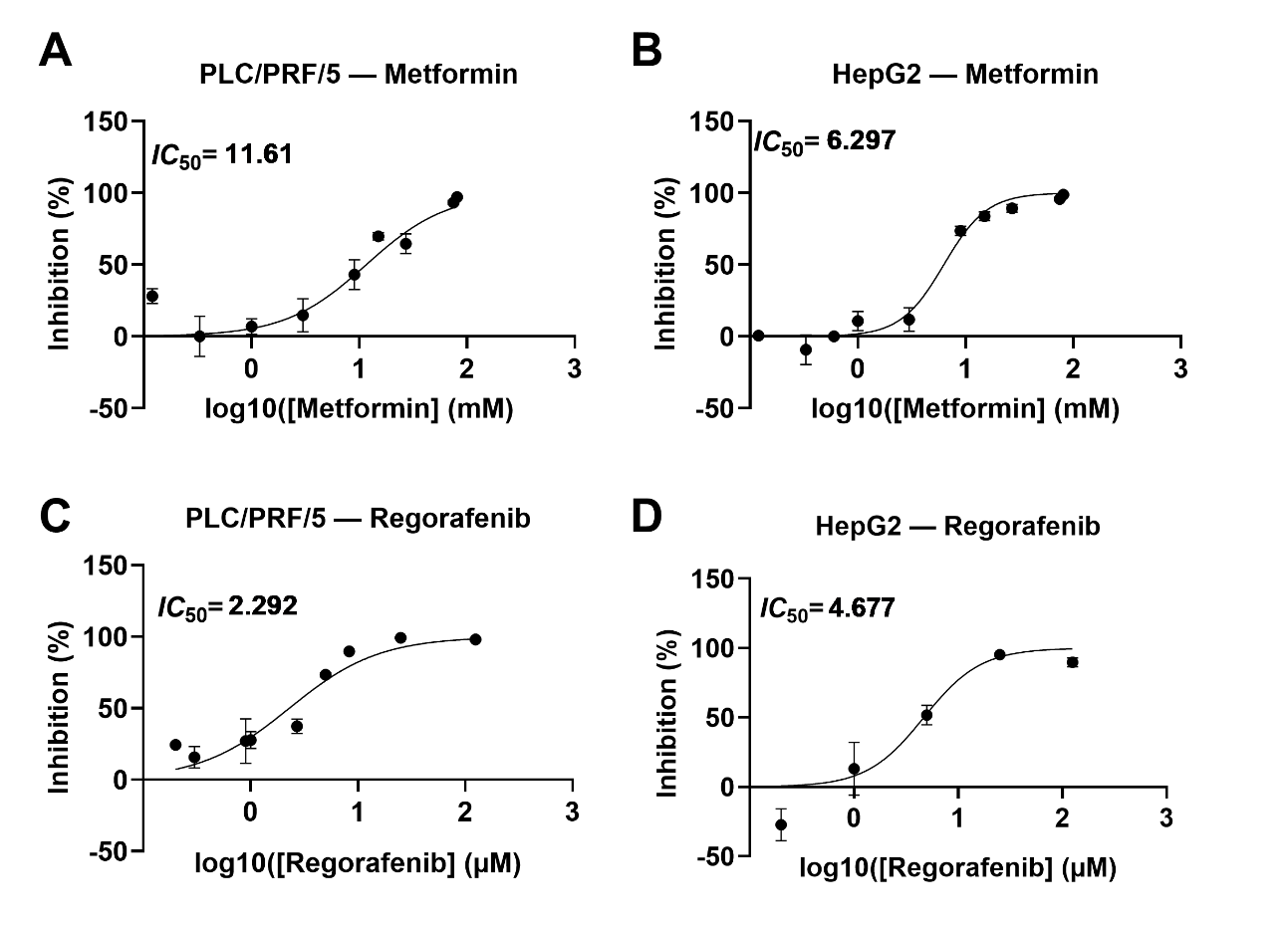


**Figure S2. Single-agent dose–response curves used to define concentration ranges for combination testing.** PLC/PRF/5 and HepG2 cells were treated for 72 h with increasing concentrations of metformin or regorafenib, and dose–response curves were fitted to estimate IC₅₀ values. Panels show: (**A**) PLC/PRF/5 metformin, (**B**) HepG2 metformin, (**C**) PLC/PRF/5 regorafenib, and (**D**) HepG2 regorafenib. The x-axis shows log10[concentration] (mM for metformin; µM for regorafenib), and the y-axis shows growth inhibition (%). Points represent mean ± SD from three independent experiments.


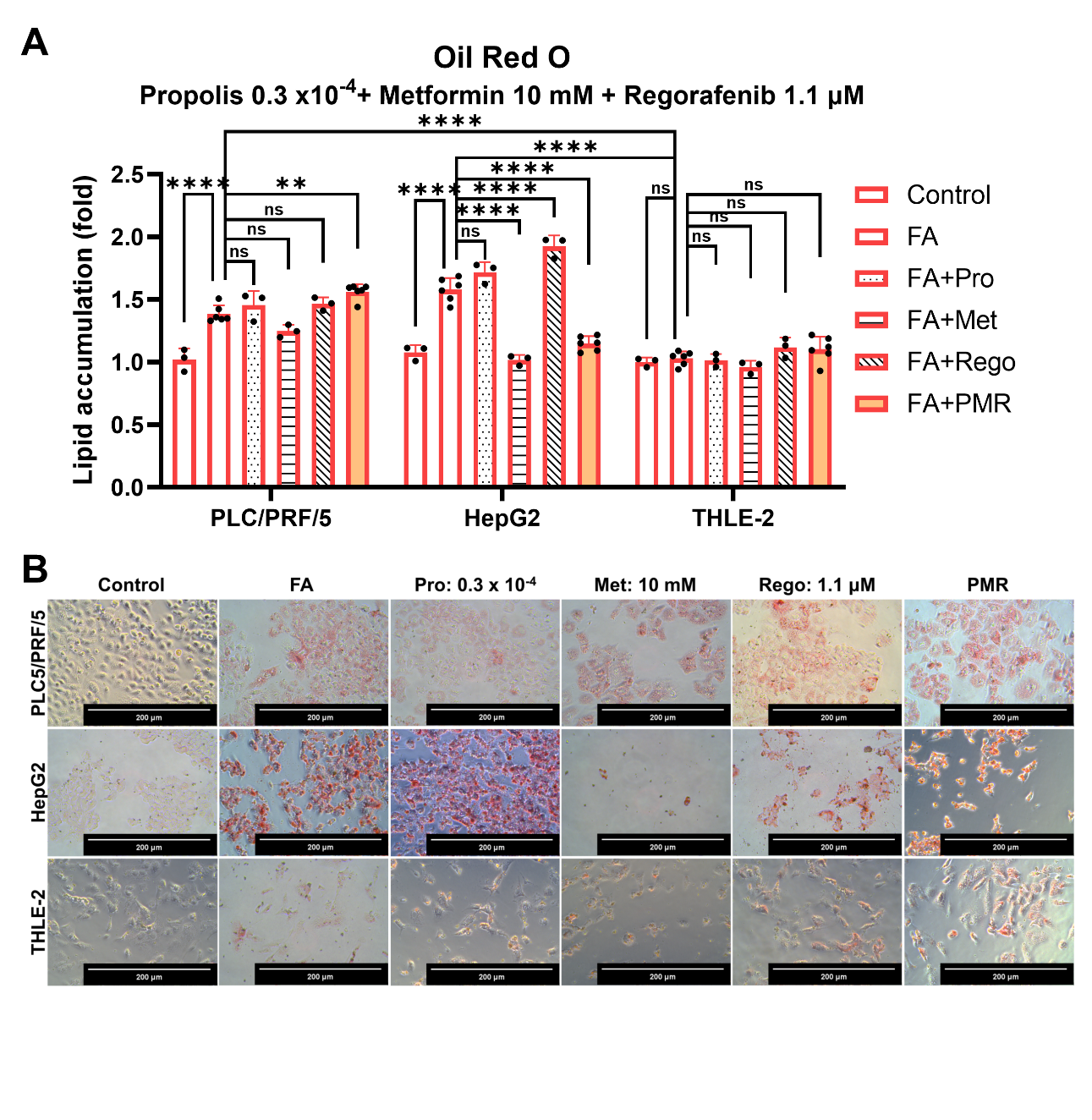


**Figure S3. Oil Red O analysis shows cell line–dependent effects of PMR on lipid accumulation under fatty acid loading.** (**A**) Quantification of Oil Red O–positive lipid accumulation (fold change relative to the untreated control) in PLC/PRF/5, HepG2, and THLE-2 cells cultured with or without fatty acids (FA) and treated with individual PMR components or the FA-optimized PMR regimen (propolis 0.3×10⁻⁴, metformin 10 mM, regorafenib 1.1 μM). Propolis is expressed as a fold-dilution of a 200 mg/mL extract stock (raw-material equivalents; 0.3×10⁻⁴ ≈ 6 μg/mL). (**B**) Representative Oil Red O micrographs for each condition (columns) in each cell type (rows). FA increased lipid accumulation in PLC/PRF/5 and HepG2, whereas THLE-2 showed comparatively limited FA-induced lipid loading. In PLC/PRF/5, PMR did not reduce Oil Red O staining relative to FA alone, while in HepG2, metformin (alone and within PMR) was associated with reduced lipid staining under FA. Scale bars, 200 μm (shown on each micrograph). Data are mean ± SD; statistical testing and significance notation follow Methods (***p* < 0.01; *****p* < 0.0001; ns, not significant).


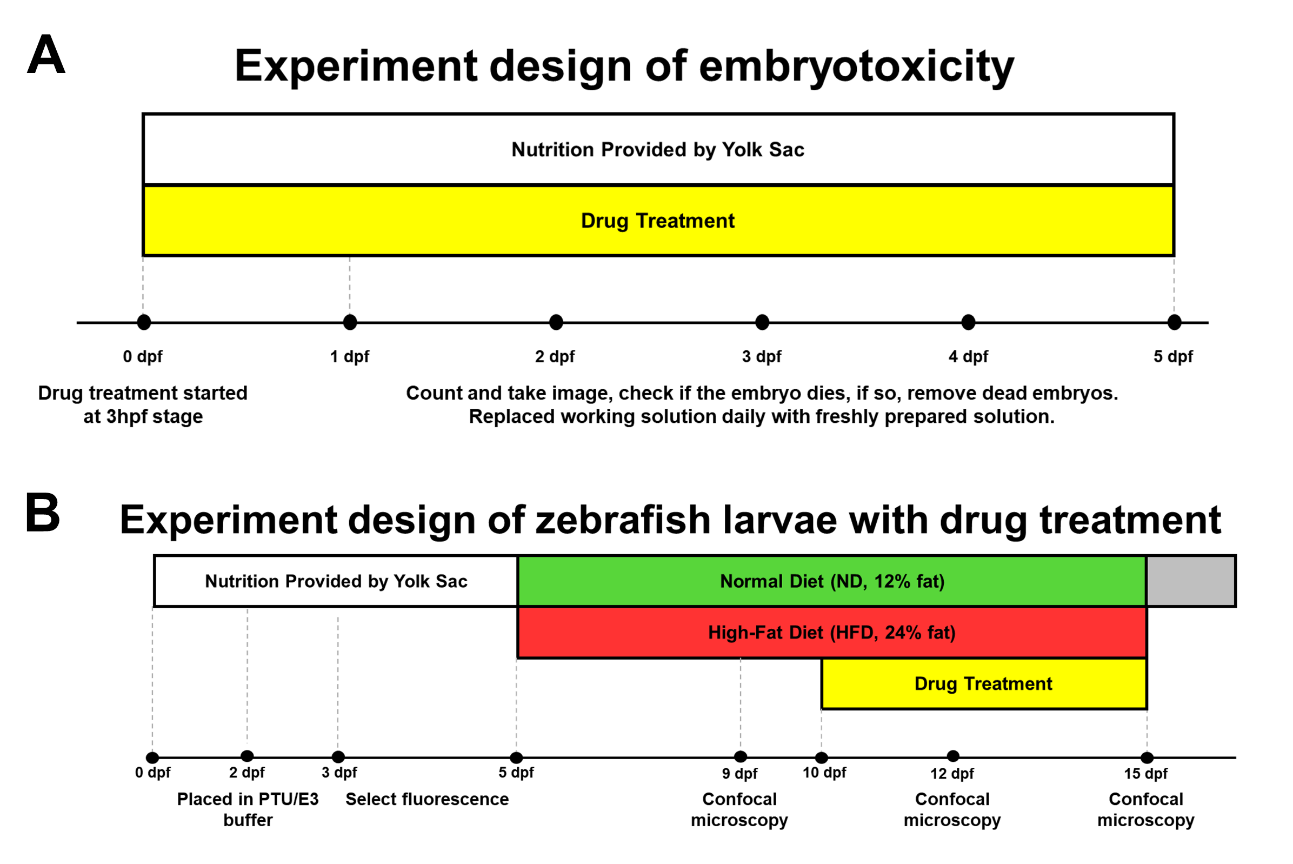


**Figure S4. Zebrafish treatment schedules for embryotoxicity and MASLD-HCC larval drug exposure.** (**A**) Embryotoxicity assay schematic. Embryos were exposed to Taiwan propolis starting at the 3 hpf stage and maintained through 5 dpf with daily replacement of freshly prepared working solutions. Embryos were monitored daily (including removal of nonviable embryos) and imaged at the indicated time points to assess developmental toxicity across the dilution series. (**B**) Larval drug-treatment timeline used in the MASLD-HCC experiments. Larvae were maintained in PTU/E3 buffer during early development, screened for fluorescence at 3 dpf, and then assigned to normal diet (ND; 12% fat) or high-fat diet (HFD; 24% fat) starting at 5 dpf. Drug treatment was administered from 10–15 dpf, with confocal imaging performed at 9, 12, and 15 dpf as indicated.


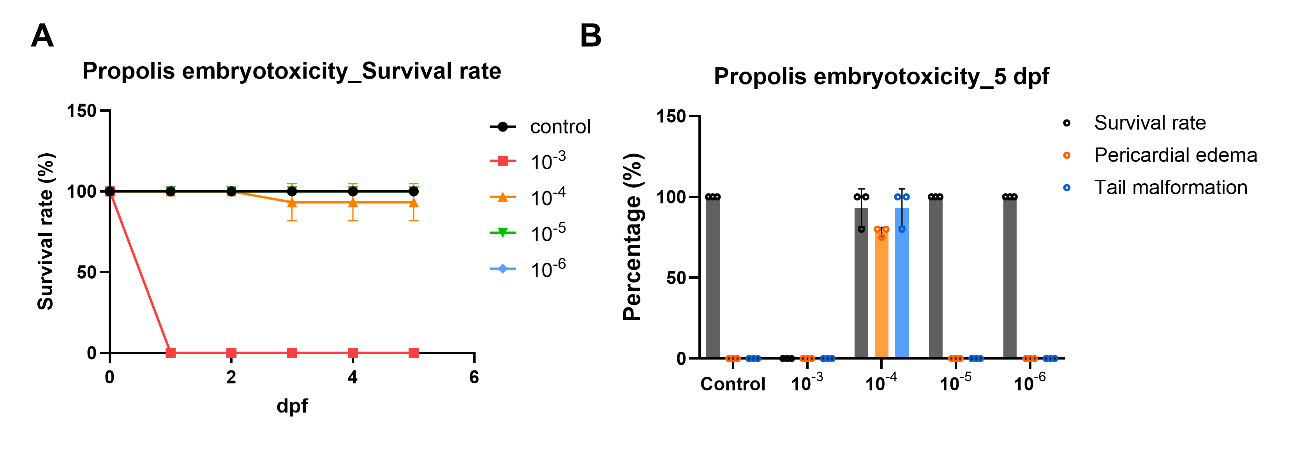


**Figure S5. Embryotoxicity profiling of Taiwan propolis in zebrafish embryos.** Tg(*mpeg:mCherry*; *mpx:EGFP*; *fabp10a:CD36*; *myl7:EGFP*); *abcg1(KO1)* embryos were exposed to Taiwan propolis across the indicated dilution series from 2 hours post-fertilization (hpf) through 5 days post-fertilization (dpf), with daily renewal of freshly prepared solutions. (**A**) Embryo survival (%) monitored longitudinally from 0–5 dpf. (**B**) Incidence (%) of developmental abnormalities scored at 5 dpf, including pericardial edema and tail malformation (shown alongside survival at 5 dpf). Data are shown as mean ± SD from independent experiments. This figure is presented descriptively to define tolerability for dose selection; no hypothesis-testing statistics or significance annotations were applied.


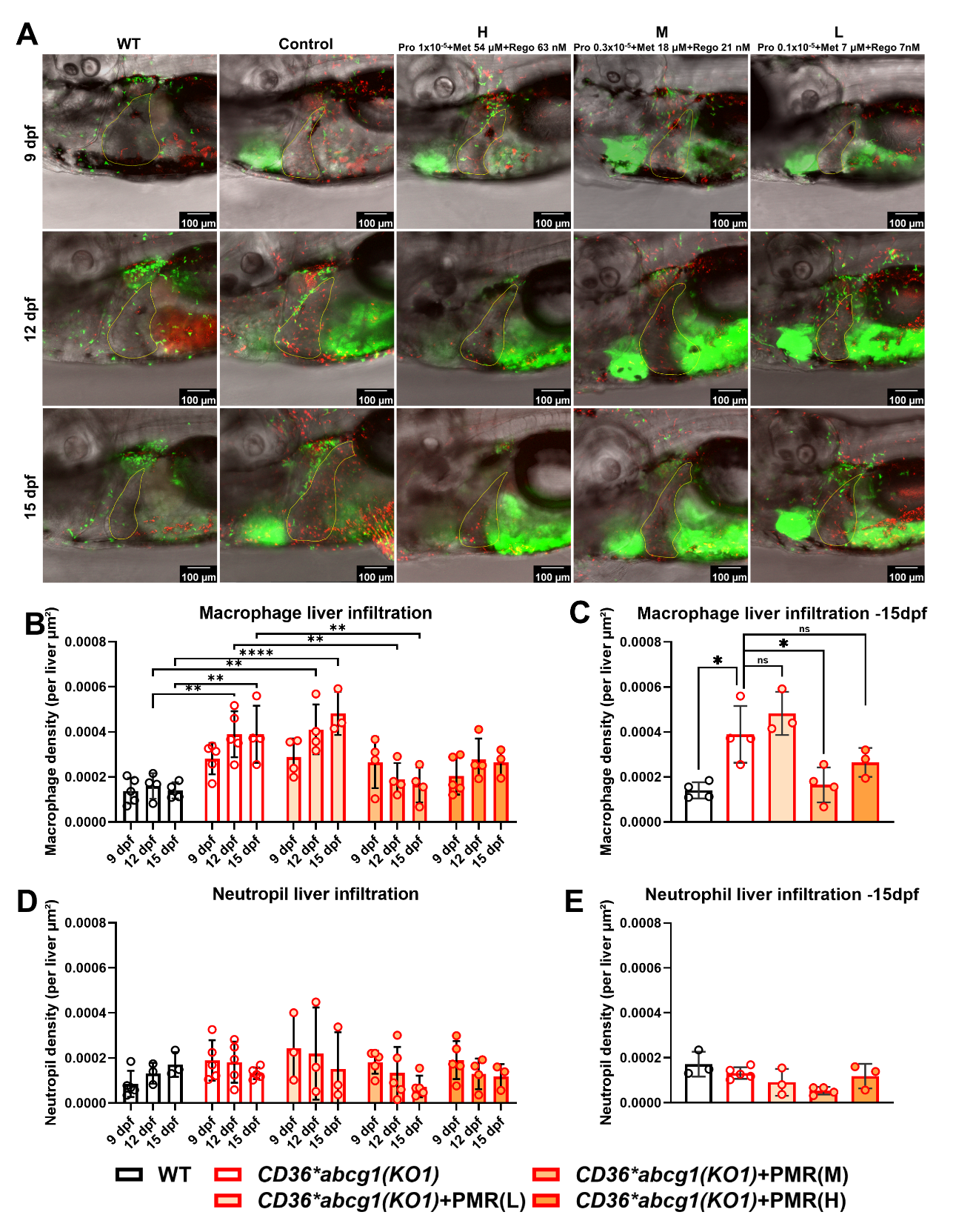


**Figure S6. PMR dose series modulates innate immune infiltration in a MASLD-HCC zebrafish model.** (**A**) Representative confocal images of the liver region (outlined) in Tg(*mpeg:mCherry*; *mpx:EGFP*) wild-type (WT) larvae and Tg(*mpeg:mCherry*; *mpx:EGFP*; *fabp10a:CD36*; *myl7:EGFP*); *abcg1(KO1)* MASLD-HCC larvae at 9, 12, and 15 dpf after PMR treatment at three dose levels: high (H; propolis 1×10⁻⁵, metformin 54 μM, regorafenib 63 nM), medium (M; propolis 0.3×10⁻⁵, metformin 18 μM, regorafenib 21 nM), or low (L; propolis 0.1×10⁻⁵, metformin 6 μM, regorafenib 7 nM). Macrophages are labeled by *mpeg:mCherry* (red) and neutrophils by *mpx:EGFP* (green); *myl7:EGFP* marks the heart as an anatomical reference. Scale bars, 100 μm (shown on each micrograph). (**B**) Area-normalized hepatic macrophage density at 9, 12, and 15 dpf. (**C**) Hepatic macrophage density at 15 dpf summarized across groups. (**D**) Area-normalized hepatic neutrophil density quantified at 9, 12, and 15 dpf. (**E**) Hepatic neutrophil density at 15 dpf summarized across groups. Immune-cell density was quantified within a predefined liver ROI and normalized to liver area (see Methods). Sample sizes are indicated in the figures as dots. Data are shown as mean ± SD. Statistical tests are described in Methods; **p* < 0.05, ***p* < 0.01, *****p* < 0.0001; ns, not significant).


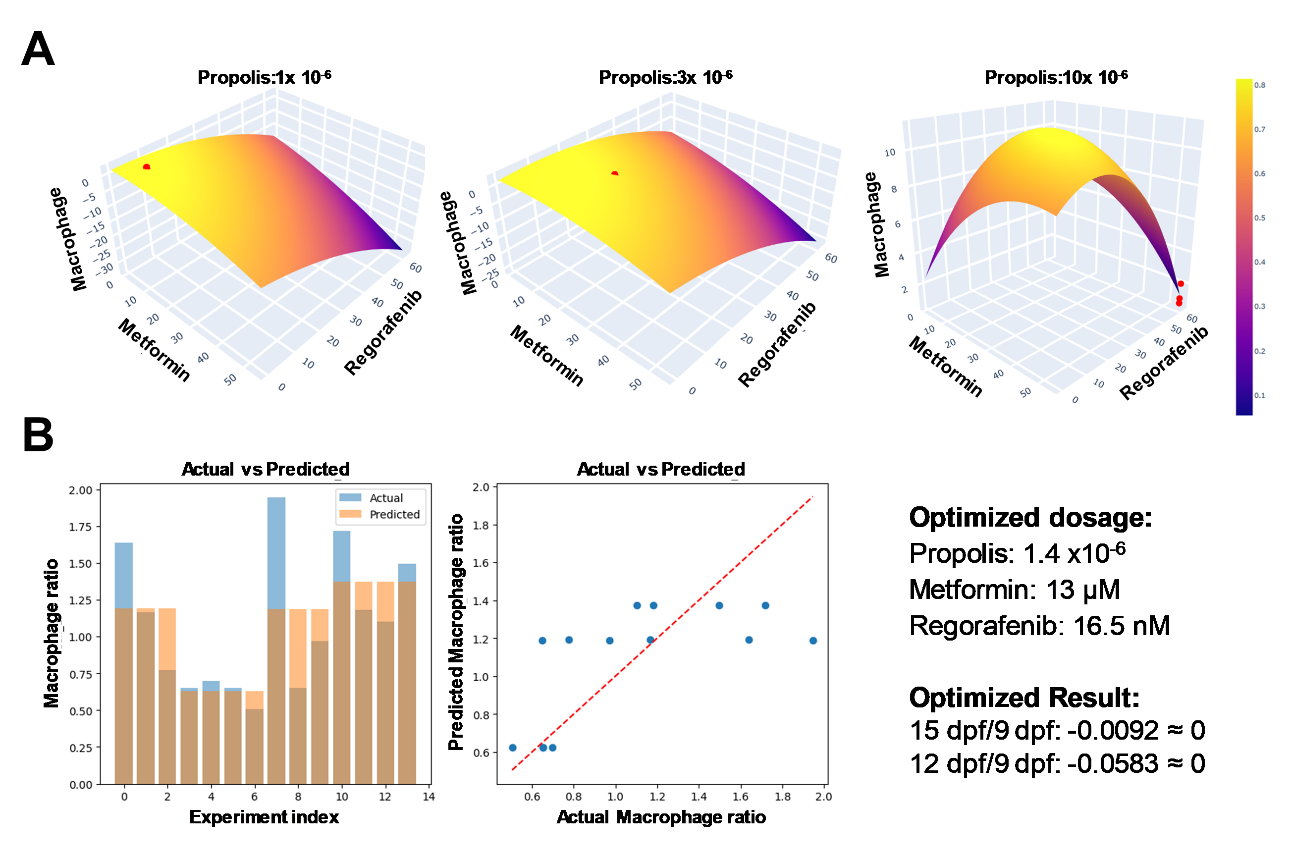


**Figure S7. PRS-OPT modeling nominates PMR doses that minimize hepatic macrophage accumulation in MASLD-HCC zebrafish.** (**A**) Phenotypic response surfaces fitted to macrophage accumulation in Tg(*mpeg:mCherry*; *mpx:EGFP*; *fabp10a:CD36*; *myl7:EGFP*); *abcg1(KO1)* larvae. The modeled outcome was the area-normalized hepatic macrophage density ratio, calculated as macrophage density at 15 dpf relative to 9 dpf (15/9); where indicated, the 12/9 ratio was also modeled. Surfaces are displayed at fixed propolis dilutions across the tested metformin–regorafenib dose space. Red points indicate experimental design points used for model fitting. (**B**) Model performance shown by observed versus predicted macrophage density ratios (summary bar plot and scatter plot). The PRS-OPT–nominated PMR dose combination and its corresponding predicted macrophage-density ratio(s) are shown at right.


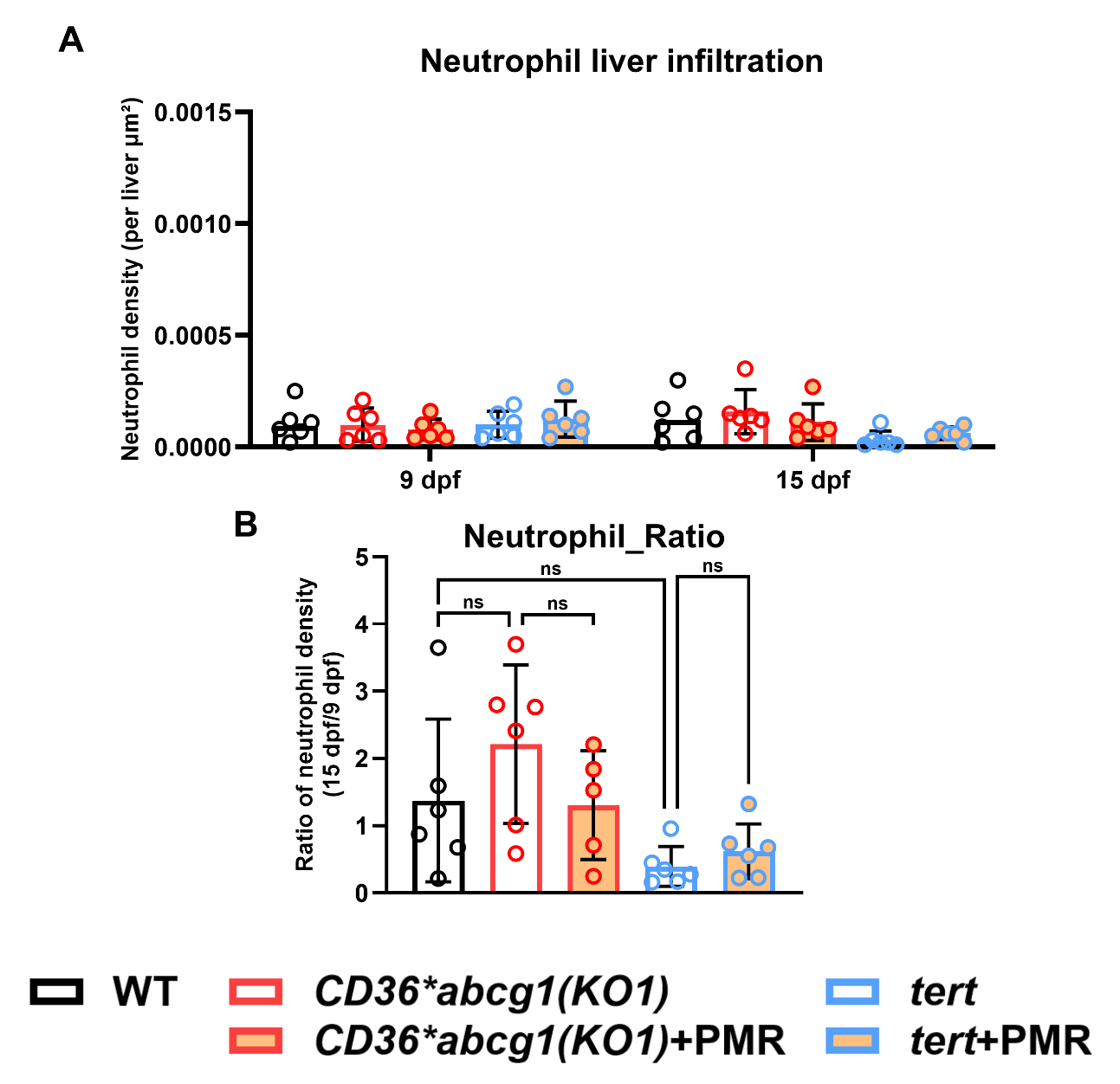


**Figure S8. PMR does not measurably alter hepatic neutrophil infiltration in zebrafish HCC models.** (**A**) Area-normalized hepatic neutrophil density quantified at 9 and 15 dpf in WT, MASLD-HCC *Tg(mpeg:mCherry; mpx:EGFP; fabp10a:CD36; myl7:EGFP); abcg1(KO1)*, and *Tg(mpeg:mCherry; mpx:EGFP; fabp10a:tert; myl7:EGFP)* larvae, with or without PRS-OPT–nominated PMR treatment. Neutrophils were quantified within a predefined liver ROI and normalized to liver area (see Methods). (**B**) Neutrophil infiltration dynamics summarized as the 15 dpf/9 dpf area-normalized neutrophil-density ratio. Points represent individual larvae; bar show mean ± SD. Statistical comparisons are indicated on the plots (ns, not significant; tests as described in Methods).


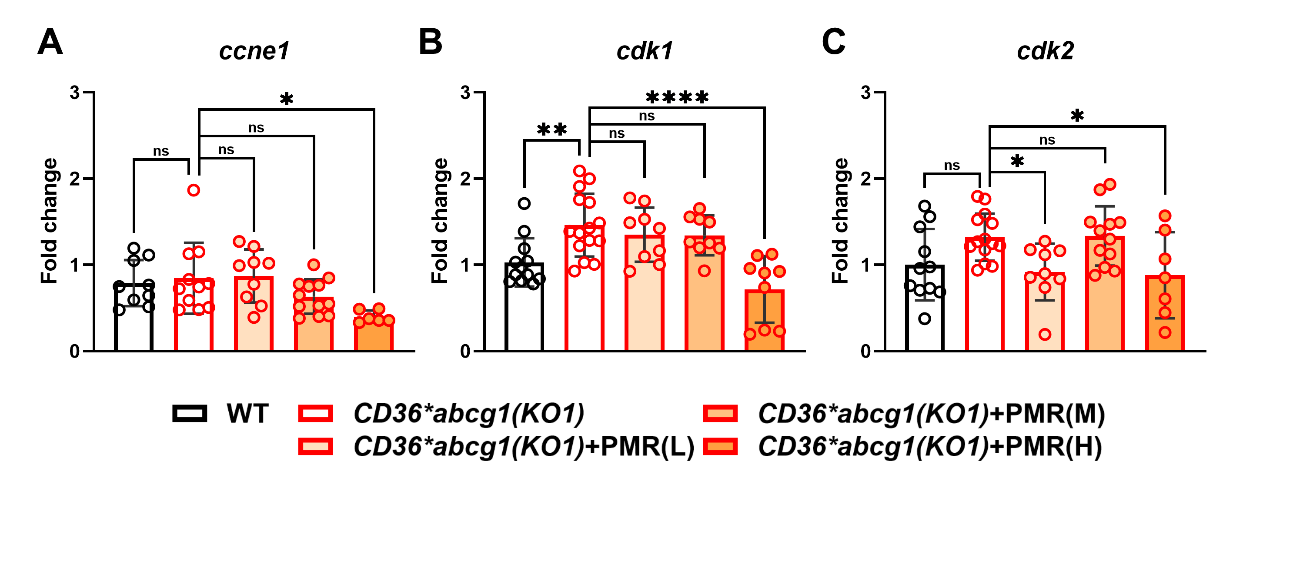


**Figure S9. PMR dose series is associated with reduced expression of cell-cycle genes in the MASLD-HCC zebrafish model.** (**A–C**) Relative mRNA expression of *ccne1* (A), *cdk1* (B), and *cdk2* (C) in CD36*abcg1(KO1) larvae treated with PMR at three dose levels: PMR(L) (propolis 0.1×10⁻⁵, metformin 6 μM, regorafenib 7 nM), PMR(M) (propolis 0.3×10⁻⁵, metformin 18 μM, regorafenib 21 nM), and PMR(H) (propolis 1×10⁻⁵, metformin 54 μM, regorafenib 63 nM). Expression was quantified by qPCR and plotted relative to the indicated control groups (WT and untreated CD36*abcg1(KO1) without PMR). Individual larvae are shown with bars indicating mean ± SD; statistical comparisons are annotated in the plots (**p*<0.05; ***p*<0.01; *****p*<0.0001; ns, not significant).


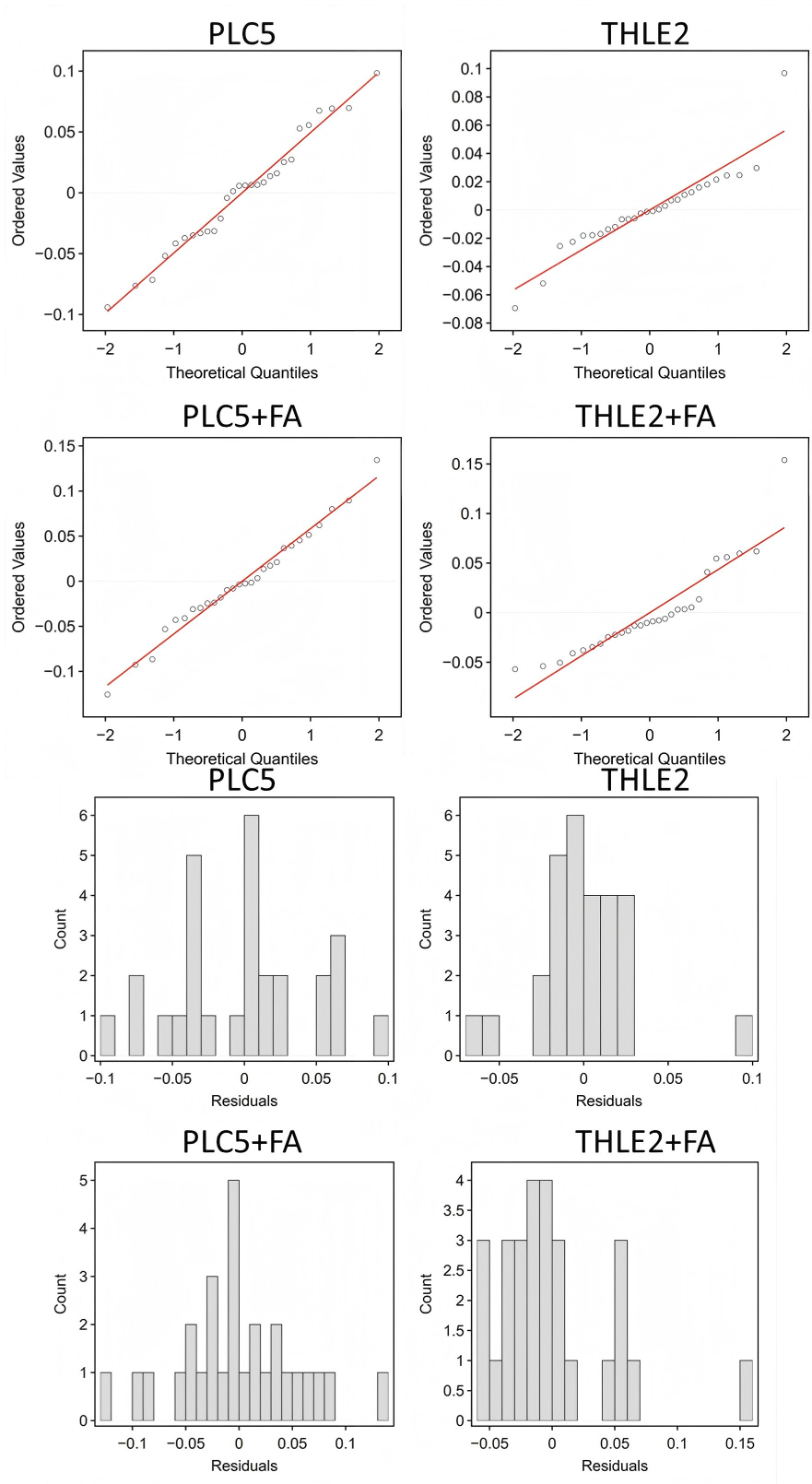


**Figure S10. Residual normality diagnostics for PRS-OPT OLS models.**

Quantile–quantile (Q–Q) plots (left) and residual histograms with kernel density overlays (right) are shown for the four quadratic OLS response-surface models fitted in this study: PLC/PRF/5 (PLC5) without fatty acids (non-FA), THLE-2 non-FA, PLC5 with fatty acids (FA), and THLE-2 FA. Residuals were defined as *e* = *y* − ŷ (observed viability minus model-predicted viability). Q–Q plots assess agreement with a theoretical normal distribution; histograms provide complementary visualization of residual distribution shape. Formal Shapiro–Wilk test results are provided in the Supplementary diagnostic summary.


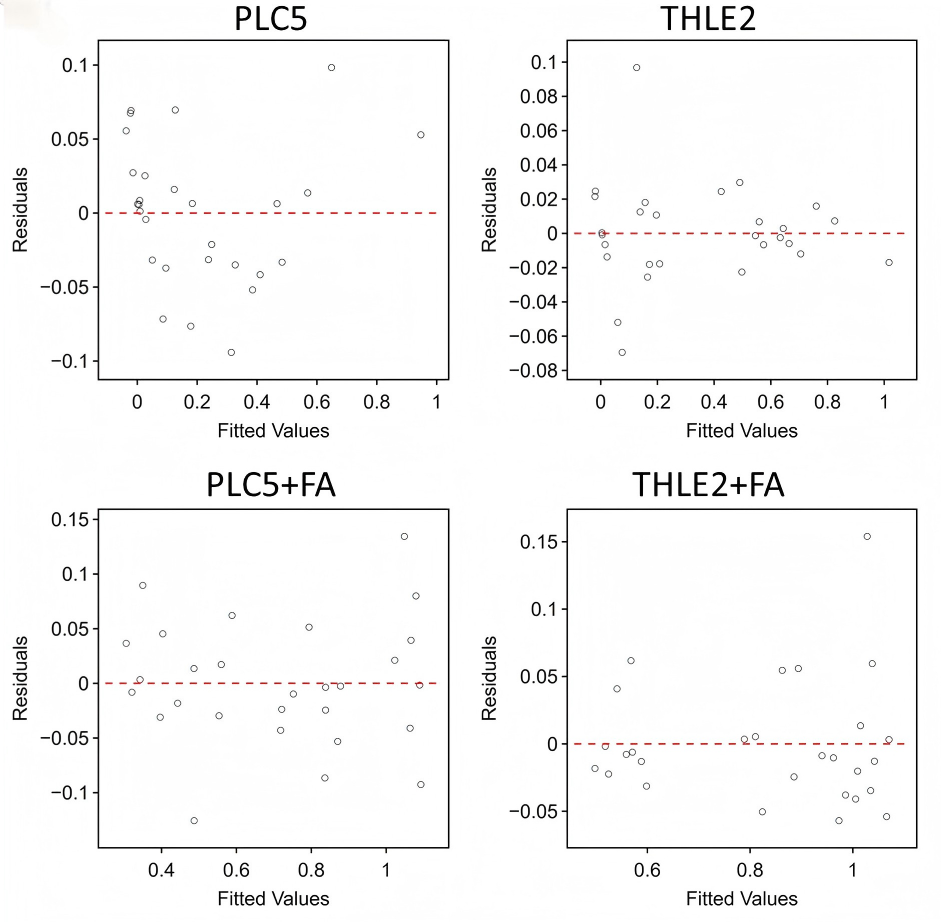


**Figure S11. Residuals versus fitted values to evaluate homoscedasticity and model form.**

Residuals (*e* = *y* − ŷ) are plotted against fitted values (ŷ) for the four PRS-OPT quadratic OLS models (PLC5 non-FA, THLE-2 non-FA, PLC5 FA, THLE-2 FA). The horizontal dashed line indicates zero residual. Visual patterns (e.g., funnel-shaped spread) were used to screen for heteroscedasticity and potential model misspecification; Breusch–Pagan tests are reported in the Supplementary diagnostic summary.


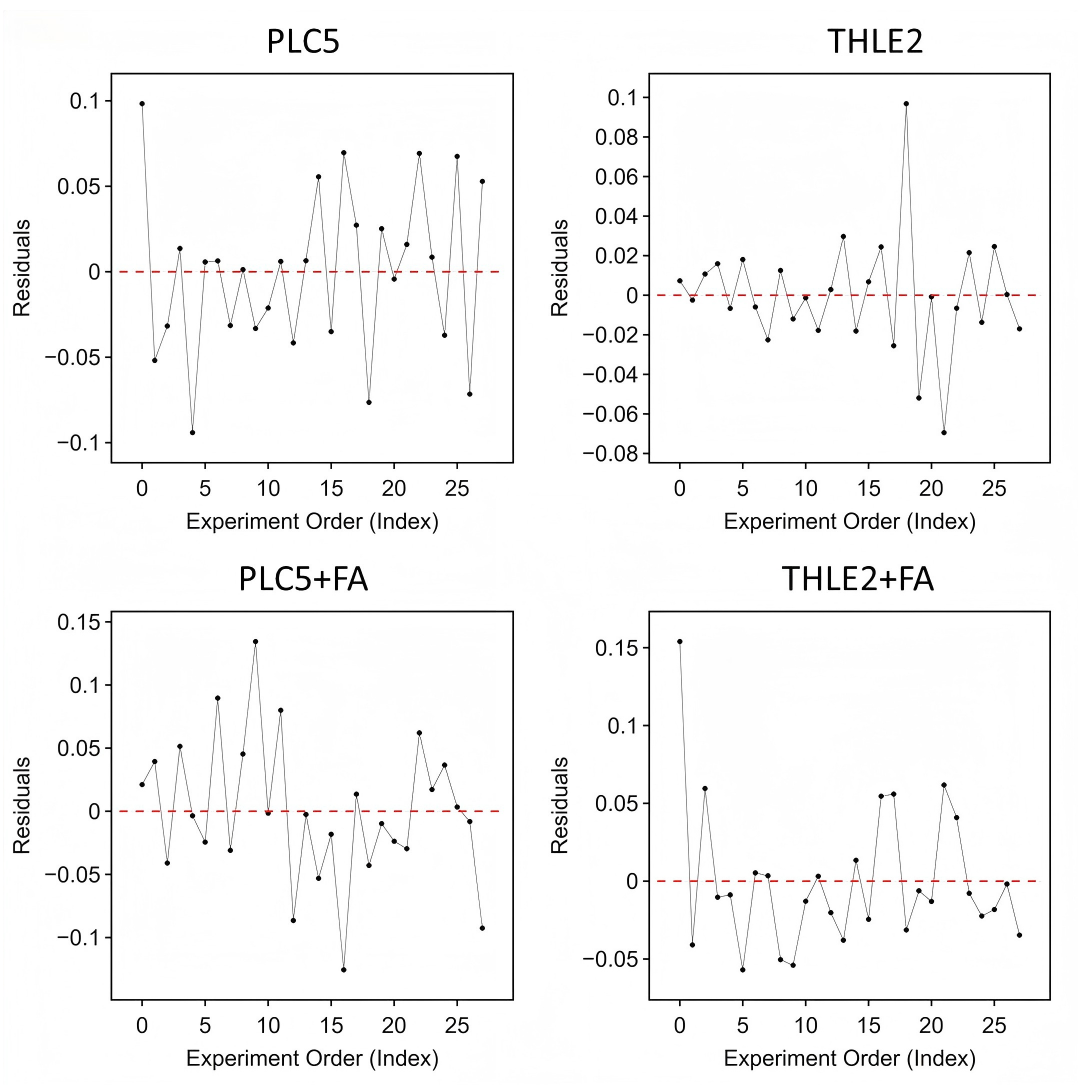


**Figure S12. Residuals versus experiment order to assess error independence.**

Residuals (*e* = *y* − ŷ) are plotted against experiment/run order for the four PRS-OPT quadratic OLS models (PLC5 non-FA, THLE-2 non-FA, PLC5 FA, THLE-2 FA). This diagnostic screens for temporal or batch-related structure in errors (autocorrelation). Durbin–Watson statistics are provided in the Supplementary diagnostic summary.
